# Polyketides isolated from an endophyte *Penicillium oxalicum* 2021CDF-3 inhibit pancreatic tumor growth

**DOI:** 10.3389/fmicb.2022.1033823

**Published:** 2022-09-26

**Authors:** Wenya Weng, Ruidian Li, Yanxia Zhang, Xiaofu Pan, Shicui Jiang, Chuchu Sun, Chi Zhang, Xuemian Lu

**Affiliations:** ^1^The Third Affiliated Hospital of Wenzhou Medical University, Zhejiang, China; ^2^Department of Endocrinology, Ruian People’s Hospital, Zhejiang, China; ^3^Shandong Research Center of Engineering and Technology for Safety Inspection of Food and Drug, Shandong Institute for Food and Drug Control, Jinan, China

**Keywords:** polyketides, secondary metabolites, algal-derived fungus, *Penicillium oxalicum*, cytotoxic activity

## Abstract

Fungal secondary metabolites are inherently considered valuable resources for new drugs discovery. To search for novel fungal secondary metabolites with lead compounds potential, a fungal strain *Penicillium oxalicum* 2021CDF-3, an endophyte of the marine red algae *Rhodomela confervoides*, was chemically studied. Cultivation of this fungus on solid rice medium yielded 10 structurally diverse metabolites (**1**–**10**), including two new polyketides, namely oxalichroman A (**1**) and oxalihexane A (**2**). Their structures were determined by detailed analysis of NMR and HRESIMS spectroscopic data. Oxalihexane A (**2**) was elucidated as a novel polyketide formed by a cyclohexane and cyclohexanone moiety *via* an ether bond. The stereochemistry of **2** was successfully assigned by NMR and ECD calculations. In the cytotoxic assay, the new compound **2** showed remarkable inhibitory effect on the human pancreatic cancer PATU8988T cell line. Further pharmacological study demonstrated that the expression level of Cyclin D1 was down-regulated by the treatment with **2**, which suggested that cell cyclin abnormity was involved in pancreatic tumor cell apoptosis. Moreover, the activation of Wnt5a/Cyclin D1 signaling pathway might be involved in the mechanism of panreatic tumor cell apoptosis induced by **2**.

## Introduction

Filamentous fungi are well known for their capability to afford tremendous bioactive molecules, termed secondary metabolites, which possess not only diverse structures but also remarkable functions (Li et al., 2021). Although some of secondary metabolites are mycotoxins and phytotoxins that tend to be problematic for humans, foods, and crops, fungal secondary metabolites have proven to be an important source of bioactive natural products with potential pharmaceutical and/or agricultural applications (Bills and Gloer, 2016). The discovery of penicillin as the first broad-spectrum antibiotic agent by Alexander Fleming in 1928 considered the “wonder drug” of World War II and then started the “Golden Age of Antibiotics” in the last century (Keller, 2019; Zhang et al., 2020). Subsequently, fungal secondary metabolites have attracted more and more attention due to their rich biological functionality and drugability (Greco et al., 2019; Keller, 2019; Shankar and Sharma, 2022). The intrinsic properties of fungal secondary metabolites make the study of these natural compounds of great significance (Hautbergue et al., 2018). Newman and Cragg revealed that 40% of all approved therapeutic agents from 1981 to 2019 were of natural origin and a significant number of natural product-derived drugs/leads are actually of microbial origin (Newman and Cragg, 2020). It should be noted that fungal secondary metabolites have become the nonnegligible source of many important approved pharmaceuticals, such as cephalosporin, griseofulvin, compactin, ergotamine, and echinocandin, with a variety of mechanisms of action (González-Medina et al., 2017). Therefore, in-depth exploration of fungal secondary metabolites with remarkable biological activities is an important approach for new drug discovery.

The genus *Penicillium* has been well-studied due to their high biosynthetic potential for producing bioactive secondary metabolites (Koul and Singh, 2017; Zhang et al., 2020). Our preliminary screening on the in-house fungi library afforded a targeted fungal strain, *Penicillium oxalicum* 2021CDF-3, which was isolated as an endophyte of the marine red algae *Rhodomela confervoides*. Initial cytotoxic assay of the EtOAc crude extracts of this strain revealed a certain inhibitory effect on various human tumor cell lines (Supplementary Table S1 in Supplementary Material), especially for the human pancreatic cancer PATU8988T cell line, with the inhibition rate of 83% at the concentration of 40 μg/ml. The above screening results indicated that this fungal strain may possess high biosynthetic potential to produce cytotoxic secondary metabolites. In order to characterize these active ingredients, a large-scale fermentation was conducted. Cultivation of this fungus on solid rice medium and further chromatographic separation yielded 10 structurally diverse polyketides (**1**–**10**), including two new ones, namely, oxalichroman A (**1**) and oxalihexane A (**2**). Their chemical structures were determined by a detailed analysis of NMR and HRESIMS spectroscopic data. Structurally, the new polyketide, oxalihexane A (**2**), was characterized as a novel polyketide formed by a cyclohexane and cyclohexanone moiety *via* an ether bond. The species *P. oxalicum* is a well-known producer of structurally diverse secondary metabolites, including chromones (Sun et al., 2012), *N*-containing alkaloids (Zhang et al., 2015), butyrolactones (Yuan et al., 2015), monoterpenoids (Zhao et al., 2022), phenylhydrazones, and quinazolines (Liu et al., 2020). Although polyketides such as chromones (compounds **1** and **7**), and phthalides (compounds **3**–**5**) were commonly found in *P. oxalicum*, it is the first time to report the isolation of **2** as the unique polyketide, indicating it as the characteristic secondary metabolite of *P. oxalicum* with chemotaxonomic significance. Moreover, compound **2** was found to induce apoptosis mediated by the activation of Wnt5a/Cyclin D1 signaling pathway in human pancreatic tumor cells. In the present study, we report the isolation, structural determination, and cytotoxic evaluation of these fungal metabolites.

## Materials and methods

### General experimental procedures

A JASCO P-1020 digital polarimeter (Tokyo, Japan) was used to detect optical rotations of the isolated compounds in MeOH. A Lambda 35 UV/Vis spectrophotometer (Perkin Elmer, Waltham, United States) was used to collect UV data of the isolated compounds. A scientific LTQ Orbitrap XL spectrometer (Thermo Scientific, Waltham, United States) was used to acquire HRESIMS. An Agilent DD2 500 MHz spectrometer (Agilent Technologies, Santa Clara, United States; 500 and 125 MHz for ^1^H and ^13^C, respectively) with tetramethylsilane (TMS) as an internal standard was used to obtain NMR spectra. HPLC was conducted on an Agilent 1,260 system using an RP-C18 column (5 mm, 10 × 250 mm, flow rate 2 ml/min, YMC, Kyoto, Japan) with MeOH (HPLC grade) as mobile phase. Silica gel (100–200 mesh and 200–300 mesh, Qingdao Marine Chemical Factory, Qingdao, China), octadecylsilyl (ODS) reversed-phase gel (30–50 μm, YMC CO., LTD., Japan), and Sephadex LH-20 (GE Healthcare, United States) were used for chromatographic separation.

### Fungal material

**Figure 1:**
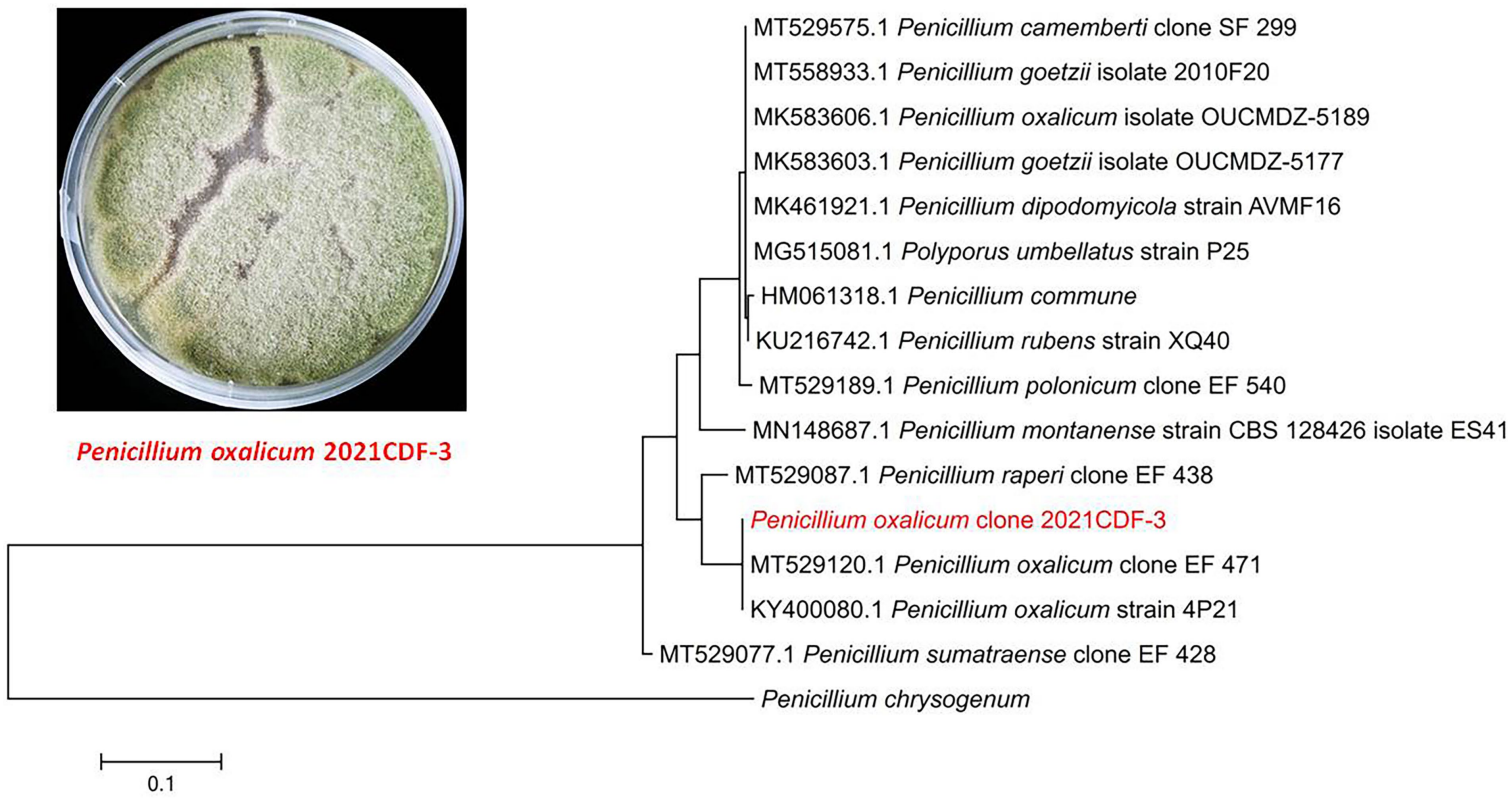
Morphology of *Penicillium oxalicum* 2021CDF-3 on PDA medium and neighbor-joining tree based on ITS nucleotide sequences.

The fungal strain *P. oxalicum* 2021CDF-3 was isolated from the marine red algae *Rhodomela confervoides*, which was collected from Lianyungang, Jiangsu province, China. This fungus was obtained from the inner tissue of *R. confervoides* with strict surface sterilizing procedures (suffered from 75% ethyl alcohol and 2.5% sodium hypochlorite). Therefore, this obtained fungus was considered as endophyte. The fungal strain was successfully identified by morphological character and sequencing of the internal transcribed spacer (ITS) of the rRNA locus. The ITS region was amplified using the ITS1 primer (TCCGTAGGTGAACCTGCGG). Then, the ITS sequence, which showed 99% identical to that of *P. oxalicum* (GenBank accession, KY400080.1), has been submitted to GenBank with the accession number of OP349593. To clarify the evolutionary position of the producing strain 2021CDF-3, a phylogenetic analysis based on the ITS sequence, together with those from other *Penicillium* species, has been performed. Results indicated that the strain 2021CDF-3 was located at the basal position of the whole tree with high confidence (100%, Figure 1). A voucher specimen of this fungus was stored at −80°C at School of Food and Pharmacy, Zhejiang Ocean University.

### Fermentation, extraction, and isolation

The producing strain was fermented in solid rice medium (*ca.* 100 g) that was previously sterilized by 100 ml of distilled seawater in a 500 ml Erlenmeyer flask. A total of 50 flasks were fermented statically with natural conditions (room temperature and sunlight) for 40 days. Afterwards, the wole cultures were extracted with EtOAc for three times. Then the EtOAc solution was collected and evaporated to dryness, which finally gave 22.6 g of brown extracts.

The extracts were subjected to open silica gel vacuum liquid chromatography column (CC, 15 × 6 cm i.d.), using mixed solvents in a gradient of increasing polarity (CH_2_Cl_2_-MeOH mixed system, from 100:1 to 10:1, v/v). Six fractions in total were obtained. Fraction 2, which was eluted with CH_2_Cl_2_-MeOH 80:1, was afforded to silica gel CC (CH_2_Cl_2_-MeOH, from 80:1 to 20:1) to yield three subfractions 2.1–2.3. Compounds **9** (2.5 mg, *t*_R_ 10.5 min) and **10** (7.8 mg, *t*_R_ 14.3 min) were isolated from subfractions 2.1 and 2.2, respectively, by semi-preparative HPLC (65% MeOH-H_2_O). Compound **8** (12.0 mg) was isolated from subfraction 2.3 by Sephadex LH-20 CC (MeOH). Fraction 3, which was eluted with CH_2_Cl_2_-MeOH 60:1, was fractionated by ODS reversed-phase CC (MeOH-H_2_O, from 10 to 100%) to give five subfractions 3.1–3.5. Compound **1** (7.0 mg) was isolated from subfraction 3.2 by preparative TLC (CH_2_Cl_2_-MeOH, 20:1), while compounds **4** (11.5 mg, *t*_R_ 9.0 min) and **7** (11.2 mg, *t*_R_ 12.3 min) were isolated from subfractions 3.3 and 3.5, respectively, by semi-preparative HPLC (55% MeOH-H_2_O). Compound **2** (26.5 mg) was isolated from Fraction 4 (eluted with CH_2_Cl_2_-MeOH 40:1) by silica gel CC (CH_2_Cl_2_-MeOH, 20:1) and followed by Sephadex LH-20 CC (MeOH). Separation of Fraction 5, which was eluted with CH_2_Cl_2_-MeOH 20:1, was found to yield compounds **3** (5.8 mg) and **5** (16.2 mg) by silica gel CC (CH_2_Cl_2_-MeOH, from 30:1 to 10:1). Finally, compound **6** (7.4 mg) was obtained from Fraction 6 by preparative TLC (CH_2_Cl_2_-MeOH-acetic acid, 10:1:0.4).

Oxalichroman A (**1**): amorphous power; [*α*]^25^_D_ − 19.1 (*c* 0.10, MeOH); UV (MeOH) *λ*_max_ (log *ε*) 215 (4.05), 253 (3.62), 326 (3.20) nm; ECD (1 mg/ml, MeOH) λ_max_ (Δε) 212 (+7.40), 252 (−0.32), 274 (+0.17), 317 (−1.54), 354 (+0.44) nm; ^1^H and ^13^C NMR data, see Table 1; HRESIMS *m/z* 245.0790 [M + Na]^+^ (calcd for C_12_H_14_O_4_Na, 245.0788).

**Table 1:** ^1^H NMR (500 MHz, *δ* in ppm) and ^13^C NMR Data (125 MHz, *δ* in ppm) of 1 and 2.

Postion	Compound**1**^a^		Postion	Compound**2**^b^	
	*δ*_H_ (*J* in Hz)	*δ*_C_, type		*δ*_H_ (*J* in Hz)	*δ*_C_, type
1		192.5, C	1		205.2, C
2	2.95, d (16.6) 2.61, d (16.6)	44.0, CH_2_	2	2.89, dd (13.9, 3.8) 2.41, m	46.6, CH_2_
3		82.2, C	3	4.44, m	69.9, CH
4		159.0, C	4	2.14, m 1.71, m	28.7, CH_2_
5	6.94, d (8.4)	118.3, CH	5	2.24, m 2.03, m	34.1, CH_2_
6	7.47, dd (8.4, 2.2)	135.2, CH	6		83.3, C
7		135.2, C	7	1.48, s	20.8, CH_3_
8	7.66, d (2.2)	123.9, CH	8		130.7, C
9		119.9, C	9	2.61, m 2.15, m	31.2, CH_2_
10	4.44, d (5.2)	62.6, CH_2_	10	4.06, m	65.8, CH
11	3.55, dd (11.6, 5.4) 3.47, dd (11.6, 5.4)	66.9, CH_2_	11	1.88, m 1.76, m	29.6, CH_2_
12	1.27, s	21.4, CH_3_	12	2.50, m 2.32, m	31.8, CH_2_
10-OH	5.19, overlap		13		155.4, C
11-OH	5.19, overlap		14	2.19, s	18.0, CH_3_
			15	10.16, s	190.8, CH
			16		170.3, C
			17	2.11, s	21.2, CH_3_

a measured in DMSO-d_6._

b measured in CDCl_3_.

Oxalihexane A (**2**): colorless gum; [*α*]^25^_D_ − 42.6 (*c* 0.12, MeOH); UV (MeOH) *λ*_max_ (log *ε*) 220 (3.89), 280 (3.96), 320 (4.08) nm; ECD (0.5 mg/ml, MeOH) λ_max_ (Δε) 214 (−7.26), 241 (−2.37), 265 (−1.11), 289 (−2.28), 326 (−0.33) nm ^1^H and ^13^C NMR data, see Table 1; HRESIMS *m/z* 309.1697 [M + H]^+^ (calcd for C_17_H_25_O_5_, 309.1702).

### Computational section

Computational details were shown in Supplementary Material.

### Cytotoxic assay

#### Cell culture

The human pancreatic cancer PATU8988T cell line was purchased from Shanghai Fuheng Biotechnology Co., LTd. The cells were cultured in RPMI 1640 medium containing 10% fetal bovine serum (Gibco, Gaithersburg, MD, United States). All cells were cultured in a humidified atmosphere of 5% CO_2_ incubator at 37°C. The medium was changed every 2 days and subcultured once they reached –80% confluence. Cells were treated with the tested compounds in the dose of 40 μM for 24 h.

#### Western blot analysis

Protein lysates of the cells were prepared in RIPA buffer (Beyotime Biotechnology, China) containing protease inhibitors (Beyotime Biotechnology). Protein concentration was measured by the Bradford assay. After being diluted in loading buffer and denatured at 95°C for 5 min, the samples were separated in 10% SDS-PAGE gel followed by being transferred into nitrocellulose membranes for separation. After blocking with 5% dried non-fat milk solution for 1 h at room temperature, the membrane was incubated with these primary antibodies, including Bax, Bcl-2, MMP-3, p53, β-Catenin, Wnt5a, and Cyclin D1 (purchased from ABclone), cleaved-Caspase3 and β-Actin (purchased from Abcam). Membranes were incubated with appropriate secondary antibodies for 1 h at room temperature following three washes with Tris-buffered saline (pH7.2) containing 0.05% Tween 20. Antigen–antibody complexes were visualized with ECL substrate (Bio-Rad Laboratories).

#### Flow cytometry

Cell appoptosis was evaluated by flow cytometry using Annexin V-FITC Apoptosis Detection Kit (Beyotime Biotechnology) according to the manufacturer’s allowed to attach over night. Then the cells were treated with or without compounds at the indicated concentration for 24 h. After that, the cells were incubated with 200 ml binding buffffer and stained with Annexin V-FITC and PI in the dark for 40 min. Then, the cells were assessed by flow cytometry (Agilent, United States).

## Results and discussion

### Structural elucidation

**Figure 2:**
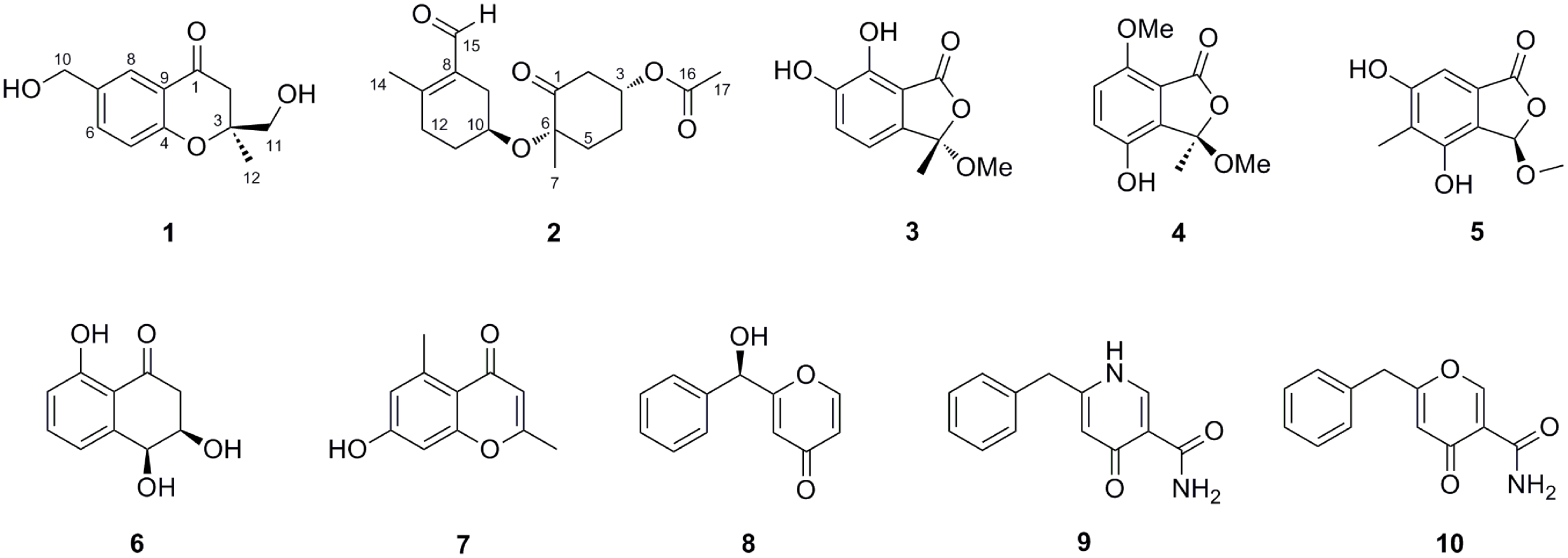
Structures of compounds 1–10.

**Figure 3:**
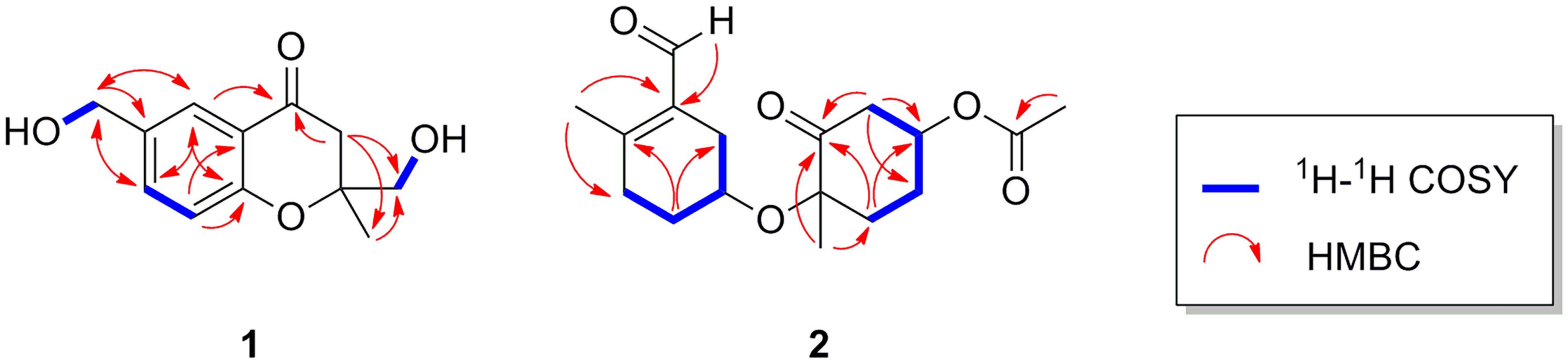
Important 1H-1H COSY and HMBC correlations of compounds 1 and 2.

**Figure 4:**
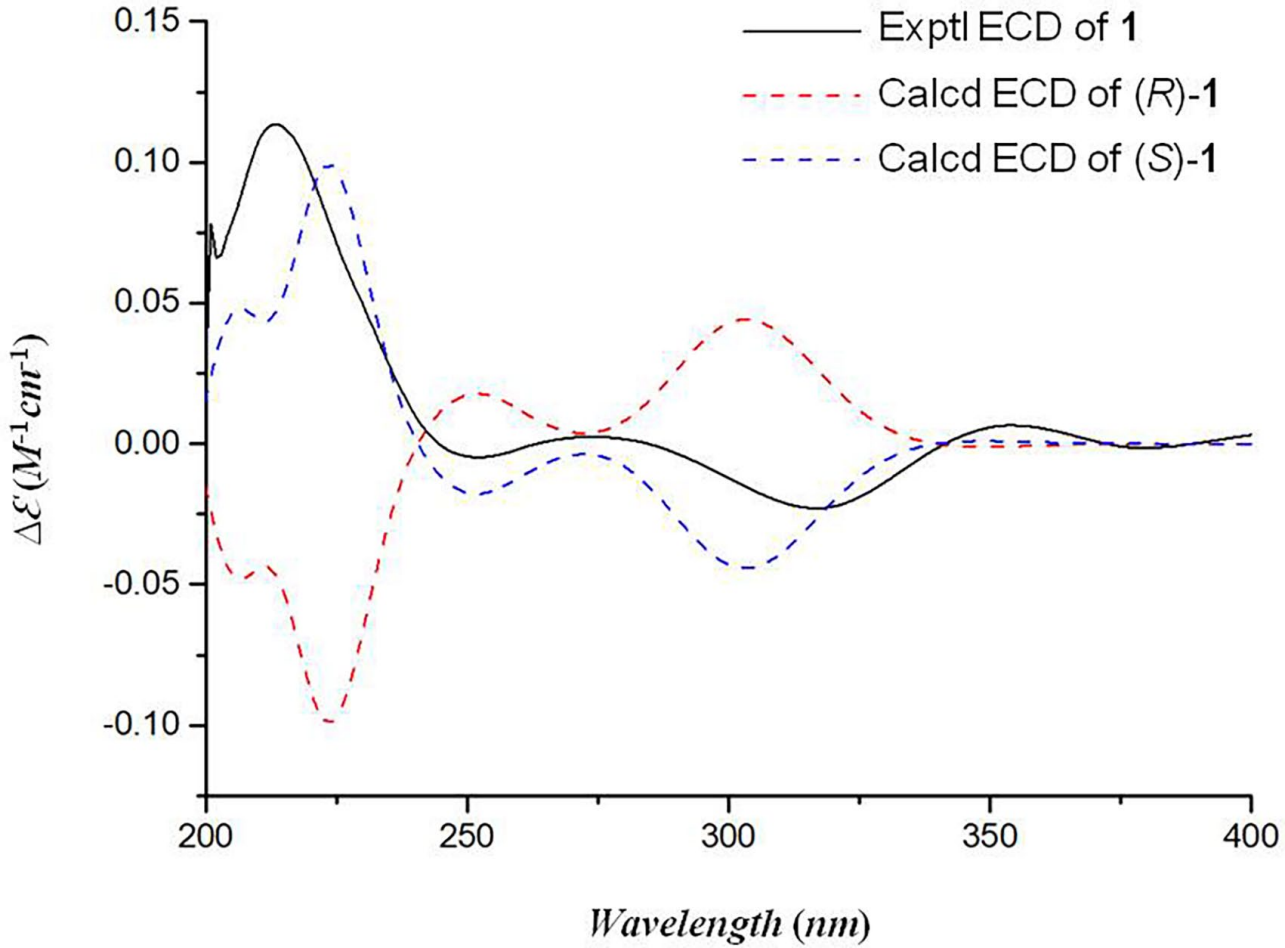
The experimental and calculated ECD spectra of compound 1.

Oxalichroman A (**1**; Figure 2) was isolated as amorphous power. Its molecular formula C_12_H_14_O_4_ was established by HRESIMS (Supplementary Figure S1 in Supplementary Material). The NMR spectra of **1** (Table 1) showed one ketone carbonyl carbon at *δ*_C_ 192.5 (C-1), signals of a 1,3,4-trisubstituted benzene ring at *δ*_C_ 118.3–159.0 (C-4 − C-9) and at *δ*_H_ 6.94 (1H, d, *J* = 8.4 Hz, H-5), 7.47 (1H, dd, *J* = 8.4, 2.2 Hz, H-6), and 7.66 (1H, d, *J* = 2.2 Hz, H-7), one oxygenated quaternary carbon at *δ*_C_ 82.2 (C-3), three methylene groups including two oxygenated at *δ*_C_ 62.6 (C-10) and at *δ*_H_ 4.44 (2H, d, *J* = 5.2 Hz, H-10), at *δ*_C_ 66.9 (C-11) and at *δ*_H_ 3.55 (1H, dd, *J* = 11.6, 5.4 Hz, H-11) and *δ*_H_ 3.47 (1H, dd, *J* = 11.6, 5.4 Hz, H-11), and one methyl group at *δ*_C_ 21.4 (C-12) and at *δ*_H_ 1.27 (3H, s, H-12). Moreover, two exchangeable OH groups were observed at *δ*_H_ 5.19 (2H, overlapped, 10-OH and 11-OH). Compound **1** possessed a benzopyrone skeleton (Kashiwada et al., 1984), which can be deduced by the key HMBC correlations from H-8 to C-1 and C-4, from H-5 to C-4 and C-9, and from H_2_-2 to C-1 (Figure 3). The location of the oxymethylene group C-10 was confirmed by the HMBC correlations from these protons to C-6, C-7, and C-8. In addition, the other oxymethylene group C-11 and the methyl group C-12 were located at C-3 due to the presence of clear HMBC correlations from H_2_-2 to C-11 and C-12, and from H_3_-12 to C-11. Thus, compound **1** was elucidated as shown in Figure 2 and was named as oxalichroman A. TDDFT calculation of the ECD spectrum of **1** at Cam-B3LYP/Def2SVP level suggested the stereochemistry of C-3 as *S*, as evidenced by the theoretical ECD curve that matched with the experimental one (Figure 4).

Oxalihexane A (**2**) was isolated as colorless gum. On the basis of the HRESIMS data, the molecular formula of **2** was determined as C_17_H_24_O_5_. Inspection of ^1^H NMR spectrum of **2** (Table 1) revealed the presence of one aldehyde group at *δ*_H_ 10.16 (1H, s, H-15), two oxygenated methine groups at *δ*_H_ 4.44 (1H, m, H-3) and *δ*_H_ 4.06 (1H, s, H-10), a set of methylene groups ranging from *δ*_H_ 1.71 to *δ*_H_ 2.89, and three methyl groups at *δ*_H_ 1.48 (3H, s, H_3_-7), 2.19 (3H, s, H_3_-14), and 2.11 (3H, s, H_3_-17). The ^13^C NMR and DEPT spectra evidenced one ketone carbonyl at *δ*_C_ 205.2 (C-1), one aldehyde group at *δ*_C_ 190.8 (C-15), one ester carbonyl at *δ*_C_ 170.3 (C-16), three methyls at *δ*_C_ 20.8 (C-7), 18.0 (C-14), and 21.2 (C-17), six methylenes (*δ*_C_ 28.7, 29.6, 31.2, 31.8, 34.1, and 46.6), two oxygenated methines at *δ*_C_ 69.9 (C-3) and 65.8 (C-10), and three quaternary carbons including two sp^2^ at *δ*_C_ 130.7 (C-8) and 155.4 (C-13) and one oxygenated sp^3^ at *δ*_C_ 83.3 (C-6). The ^1^H-^1^H COSY cross peaks of H_2_-9/H-10/H_2_-11/H_2_-12 constructed a − CH_2_CHCH_2_CH_2_− spin system (Figure 3). Further important HMBC correlations, including HMBCs from H_3_-14 to C-8 and C-12, from H_2_-11 to C-9 and C-13, and from H-15 to C-8 (Figure 3) indicated the presence of a cyclohexane moiety. Moreover, COSY correlations between H_2_-2/H-3, H-3/H_2_-4, and H_2_-4/H_2_-5 and key HMBCs from H_2_-2 to C-1, from H_2_-5 to C-1 and C-3, and from H_3_-7 to C-1 and C-5 revealed a cyclohexanone moiety (Figure 3). The above cyclohexane and cyclohexanone moieties were connected *via* an ether bond based on detailed analysis of HRESIMS and chemical shifts of C-6 and C-10. In addition, the acetyl group was attached to C-3 based on the HMBC correlation from H-3 to C-16. The structure of **2** was thus determined accordingly.

**Figure 5:**
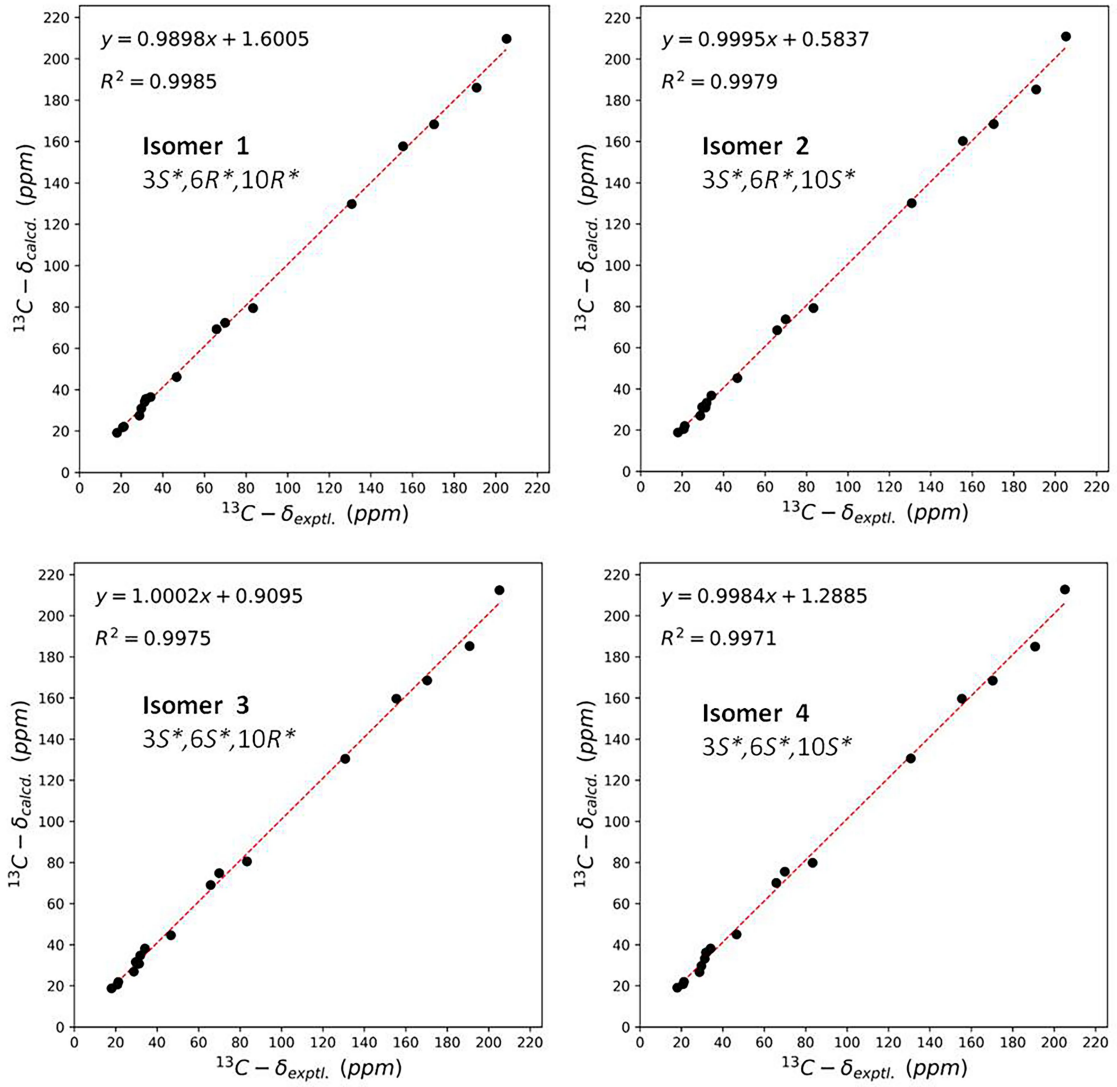
Linear regression analysis between the experimental and calculated NMR data of conformers of isomer 1–isomer 4.

**Figure 6:**
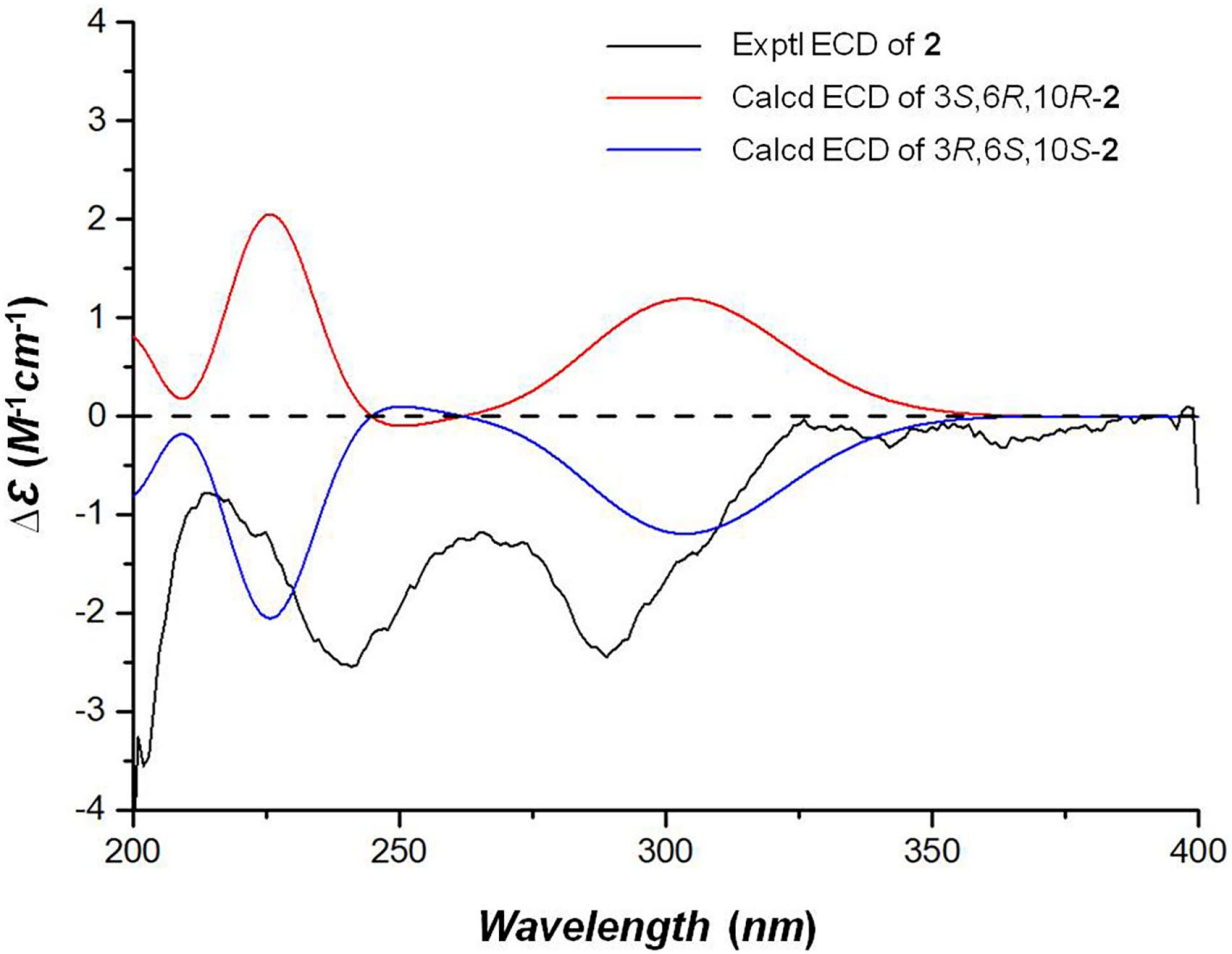
The experimental and calculated ECD spectra of compound 2.

The NOE correlations gave useless information to determine the relative configuration of **2** (Supplementary Figure S13 in Supplementary Material). To establish the relative stereochemistry of **2**, (3S*,6R*,10R*)-**2**, (3S*,6R*,10S*)-**2**, (3S*,6S*,10R*)-**2**, and (3S*,6S*,10S*)-**2** were subjected to quantum chemical calculation of chemical shifts under the theory level of MPW1PW91-SCRF/6–31 + G(d,p)//B3LYP/6-31G(d) with the IEFPCM solvent model. As a result, the calculated ^13^C NMR data of (3S*,6R*,10R*)-**2** were found to be in better agreement with their experimental counterparts, as indicated by *R*^2^ and supported by DP4+ probability analysis (Figure 5). Thus, the relative configuration of **2** was assigned as 3S*,6R*,10R*, and subsequent TDDFT ECD calculation at the Cam-B3LYP/Def2SVP, which was run on one of the two possible enantiomers, (3*S*,6*R*,10*R*)-**2** and (3*R*,6*S*,10*S*)-**2**, succeeded in the establishment of the absolute configuration of **2** as 3*R*,6*S*,10*S* (Figure 6).

In addition, eight previously reported compounds (**3**–**10**) were also isolated from this fungus. They were finally characterized as 6,7-dihydroxy-3-methoxy-3-methylphthalide (**3**) (Wang et al., 2013), chrysoalide B (**4**) (Ge et al., 2021), rubralide C (**5**) (Kimura et al., 2007), *cis*-(3*RS*,4*SR*)-3,4-dihydro-3,4,8-trihydroxynaphthalen-1(2*H*)-one (**6**) (Couché et al., 2009), 2,5-dimethyl-7-hydroxychromone (**7**) (Kashiwada et al., 1984), (7*R*)-(hydroxy(phenyl)methyl)-4*H*-pyran-4-one (**8**) (Xu et al., 2019), 6-benzyl-4-oxo-1,4-dihydropyridine-3-carboxamide (**9**) (Ye et al., 2005), and carbonarone A (**10**) (Zhang et al., 2007), respectively, by comparison of their spectroscopic data with literatures.

### Cytotoxic activity

**Figure 7:**
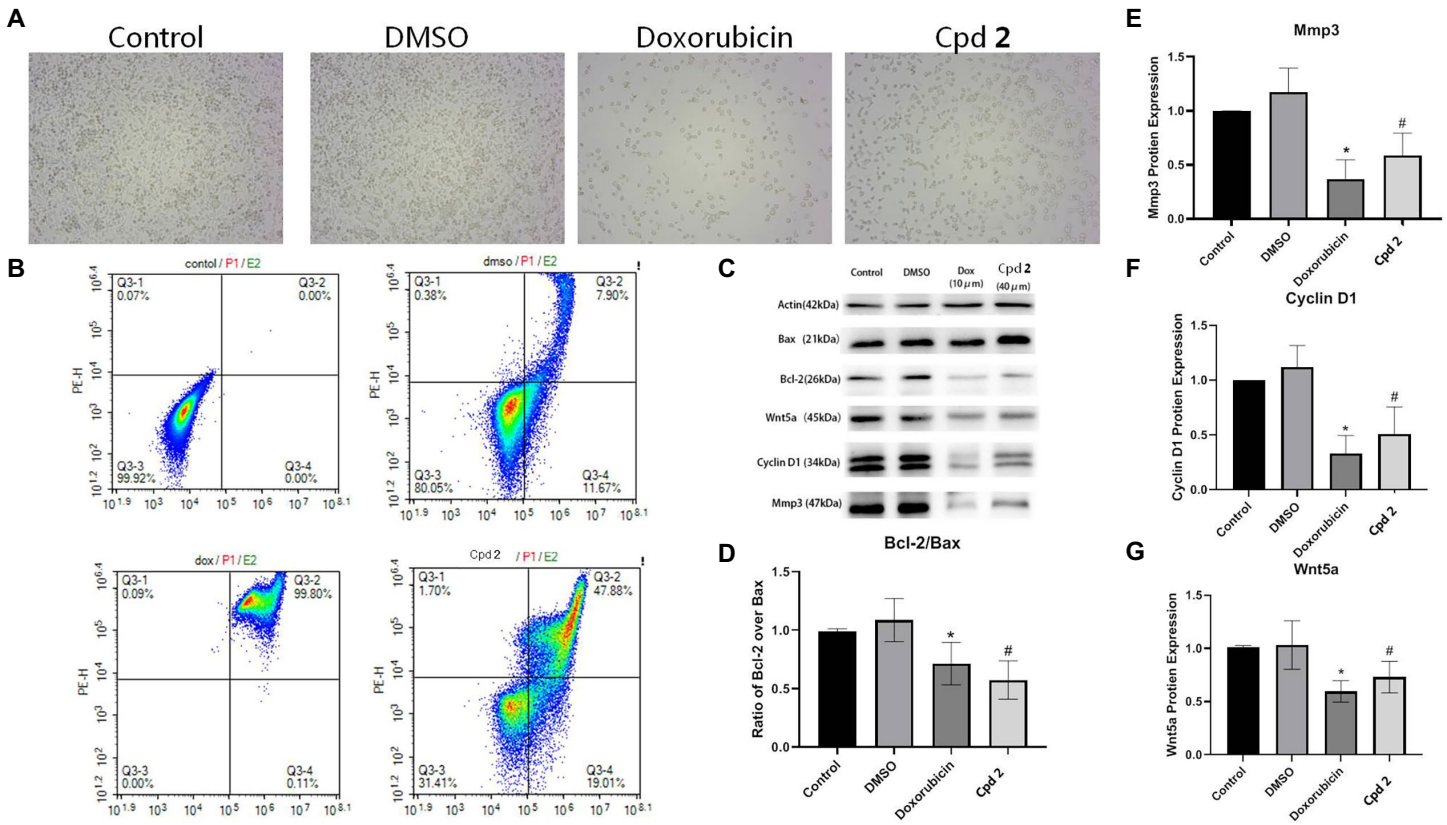
Compound **2** (Cpd **2**) alleviated the apoptosis induced in human pancreatic cancer cells. **(A)** Morphological changes of human pancreatic cancer cell (PATU8988T). **(B)** Functions of **2** in cell apoptosis in human pancreatic cancer cell (PATU8988T). Annexin V/PI double staining with flow cytometry analysis was applied for cell apoptosis. **(C)–(G)** Western blotting of Bcl-2/Bax, Mmp3, Cyclin D1 and Wnt5a. *p < 0.05, doxorubicin group vs. DMSO group; ^#^*p* < 0.05, test group vs. DMSO group.

The new compounds **1** and **2** were evaluated for their cytotoxicity against the human pancreatic cancer PATU8988T cell line. Compound **2** was found to possess promising activity with the inhibition rate of 93% at the concentration of 20 μM. In order to explore whether the proliferation inhibition of PATU8988T cells was related to the cell apoptosis, we detected apoptosis indicators. After the cells were treated with **2** at the concentration of 40 μM for 24 h, cell number reduction and cell morphology abnormity including pyknosis, shrinkage and dissociated from the plate were observed in both doxorubicin and **2** treated groups under a light microscope. While in contrast, the cells in the control group grew well (Figure 7A), suggesting compound **2** as well as doxorubicin might induce tumor cell death. In addition, Annexin V-FITC/PI assay was performed to detect apoptosis percentatge by flow cytometry. As shown in Figure 7B, both doxorubicin and **2** remarkbly increased the proportion of apoptotic cells. Additionally, western blotting was applied to further detect whether apoptosis related indicators were altered in the cells treated with **2**. As shown in Figures 7C,D **2** significantly down-regulated the ratio of Bcl-2/Bax, indicating that cell apoptosis occurred after treated with **2**. In addition, we also detected the expression level of MMP-3. Figures 7C,E showed that **2** decreased the expression level of MMP-3, a tumor indicator. To investigate whether Wnt5a/Cyclin D1 pathway was involved in the **2**-induced apoptosis, the expression levels of Wnt5a and Cyclin D1 in cells treated with the doxorubicin and **2** were both evaluated. The results demonstrated that the expression levels of Cyclin D1 and Wnt5a were both dramatically down-regulated by doxorubicin as well as **2** (Figures 7C,F,G). The above results suggested that compound **2** might induce the apoptosis of pancreas cancer cells through Wnt5a/Cyclin D1 signaling pathway.

## Conclusion

In summary, chemical examination of the endophytic fungus *P. oxalicum* 2021CDF-3 resulted in the isolation of 10 diverse polyketides. Among them, compounds **1** and **2** were characterized as new compounds. Oxalihexane A (**2**), elucidated as a novel polyketide formed by a cyclohexane and cyclohexanone moiety, showed remarkable inhibitory effect on the human pancreatic cancer PATU8988T cell line. Apoptosis is involved in the regulation of tumor cell proliferation. Compound **2** induced remarkable apoptosis in human pancreatic tumor cells, characterized by the morphologies abnormity, the decrease in cell number and the ratio of Bcl-2 to Bax, in the **2**-treated group compared with the control group. Understanding of underlying mechanism is of significance to explore more effective therapeutic strategy for pancreatic tumor treatment. In this work, the result demonstrated that the expression level of Cyclin D1 was down-regulated by **2**, suggesting that cell cyclin abnormity was involved in pancreatic tumor cell apoptosis. Furthermore, we found that the activation of Wnt5a/Cyclin D1 signaling pathway might be involved in the mechanism of pancreatic tumor cell apoptosis induced by **2**.

## Data availability statement

The datasets presented in this study can be found in online repositories. The names of the repository/repositories and accession number(s) can be found in the article/Supplementary material.

## Author contributions

WW and XL: conception or design. RL, WW, YZ, XP, SJ, and CS: acquisition, analysis, or interpretation of data. WW, XL, and CZ: drafting the work or revising. WW, CZ, and XL: final approval of the manuscript. All authors reviewed the manuscript. All authors contributed to the article and approved the submitted version.

## Funding

This study was supported by grant from the Ruian Bureau of Science and Technology (MS2022004, to WW).

## Conflict of interest

All authors declare that the research was conducted in the absence of any commercial or financial relationships that could be construed as a potential conflict of interest.

## Publisher’s note

All claims expressed in this article are solely those of the authors and do not necessarily represent those of their affiliated organizations, or those of the publisher, the editors and the reviewers. Any product that may be evaluated in this article, or claim that may be made by its manufacturer, is not guaranteed or endorsed by the publisher.
