# Supplementary material for: Polyketides isolated from an endophyte Penicillium oxalicum 2021CDF-3 inhibit pancreatic tumor growth
Source: Front Microbiol. 2022 Sep 26;13:1033823. doi: 10.3389/fmicb.2022.1033823 (PMC9549284; doi:10.3389/fmicb.2022.1033823)
Supplement: Supplementary file 1 [file Data_Sheet_1.docx]

**Supplementary Material**

Polyketides isolated from an endophyte *Penicillium oxalicum* 2021CDF-3 inhibit pancreatic tumor growth

Wenya Weng ^1, †^, Ruidian Li ^1, 2 †^, Yanxia Zhang ^3^, Xiaofu Pan ^1^, Shicui Jiang ^1^, Chuchu Sun ^1^, Chi Zhang ^1,^*, Xuemian Lu ^1, 2^*

^1^The Third Affiliated Hospital of Wenzhou Medical University, Zhejiang 325200, China

^2^Department of Endocrinology, Ruian people’s Hospital, Zhejiang 325200, China

^3^Shandong Research Center of Engineering and Technology for Safety Inspection of Food and Drug, Shandong Institute for Food and Drug Control, Jinan 250101, China.

***Correspondence:**

Xuemian Lu: luxuemian@wmu.edu.cn

Chi Zhang: zhangchi515@126.com

^†^These authors contributed equally to this work.

Keywords: Polyketides, Secondary metabolites, Algal-derived fungus, *Penicillium oxalicum*, Cytotoxic activity

**Contents**

**Figure S1.** HRESIMS spectrum of compound **1**

**Figure S2.** ^1^H NMR (500 MHz, DMSO-*d*_6_) spectrum of compound **1**

**Figure S3.** ^13^C NMR (125 MHz, DMSO-*d*_6_) spectrum of compound **1**

**Figure S4.** HSQC spectrum of compound **1**

**Figure S5.** COSY spectrum of compound **1**

**Figure S6.** HMBC spectrum of compound **1**

**Figure S7.** HRESIMS spectrum of compound **2**

**Figure S8.** ^1^H NMR (500 MHz, CDCl_3_) spectrum of compound **2**

**Figure S9.** ^13^C NMR (125 MHz, CDCl_3_) spectrum of compound **2**

**Figure S10.** HSQC spectrum of compound **2**

**Figure S11.** COSY spectrum of compound **2**

**Figure S12.** HMBC spectrum of compound **2**

**Figure S13.** NOESY spectrum of compound **2**

**Table S1** Preliminary screening results of the crude extracts of *Penicillium oxalicum* 2021CDF-3

**Computational Section**

**
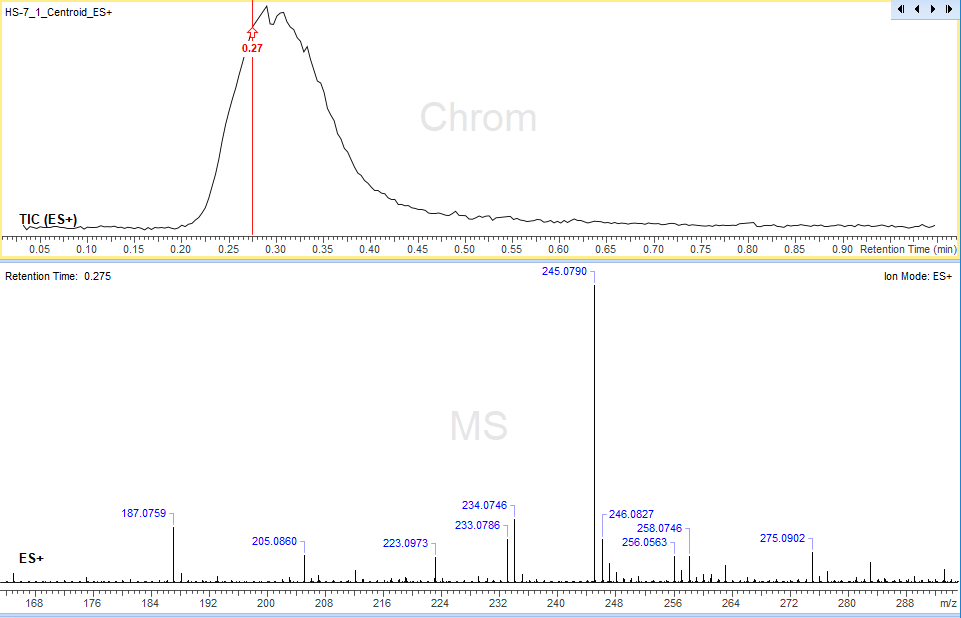
Figure S1.** HRESIMS spectrum of compound **1**

**Figure S2.** ^1^H NMR (500 MHz, DMSO-*d*_6_) spectrum of compound **1**

**Figure S3.** ^13^C NMR (125 MHz, DMSO-*d*_6_) spectrum of compound **1**

**Figure S4.** HSQC spectrum of compound **1**

**Figure S5.** COSY spectrum of compound **1**

**Figure S6.** HMBC spectrum of compound **1**

**
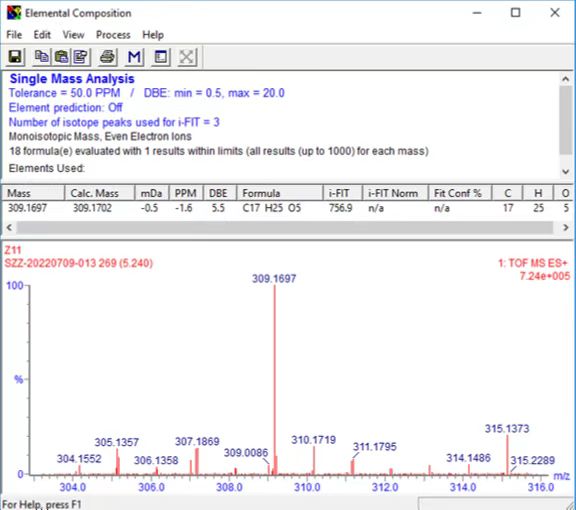
**

**Figure S7.** HRESIMS spectrum of compound **2**

**Figure S8.** ^1^H NMR (500 MHz, CDCl_3_) spectrum of compound **2**

**Figure S9.** ^13^C NMR (125 MHz, CDCl_3_) spectrum of compound **2**

**Figure S10.** HSQC spectrum of compound **2**

**Figure S11.** COSY spectrum of compound **2**

**Figure S12.** HMBC spectrum of compound **2**

**Figure S13.** NOESY spectrum of compound **2**

**Table S1** Preliminary screening results of the crude extracts of *Penicillium oxalicum* 2021CDF-3

|  | HeLa^a^ | HepG2 | A549 | PATU8988T | HCT116 | MCF7 | A2780 |
| --- | --- | --- | --- | --- | --- | --- | --- |
| crude extracts | (70±0.5)% | (66±0.9)% | (73±0.2)% | (83±0.5)% | (48±0.1)% | (26±0.2)% | (56±0.3)% |

^a^ concentration, 40 μg/mL

**Computational Section**

The conformer rotamer ensemble sampling tool (crest) [^1^](#_ENREF_1) was utilized to generate candidate conformers and DFT calculations were performed using the Gaussian 16 program [^2^](#_ENREF_2). The conformers within an energy window of 10 kcal/mol were optimized at B3LYP/6-31G(d) level of theory with Grimme's D3 dispersion correction (“EmpiricalDispersion=GD3” key words in input files). Frequency analysis of all optimized conformations was undertaken at the same level of theory to ensure they were true local minima on the potential energy surface. Then, energies of all optimized conformations were evaluated by M062X/6-311+G(2d,p) with D3 dispersion correction. Gibbs free energies of each conformers were calculated by adding “Thermal correction to Gibbs Free Energy” obtained by frequency analysis to electronic energies obtained at M062X/6-311+G(2d,p). Room-temperature (298.15 K) equilibrium populations were calculated according to Boltzmann distribution law. Those conformers accounting for over 2% population were subjected to subsequent calculations. Time-dependent density-functional theory (TDDFT) ECD calculations were run at Cam-B3LYP/Def2SVP level of theory in MeOH with IEFPCM solvent model, respectively. For each conformer, 30 excited states were calculated [^3^](#_ENREF_3). The calculated ECD curves were generated using Multiwfn 3.6 software [^4^](#_ENREF_4).

1. Pracht, P.; Bohle, F.; Grimme, S., Automated exploration of the low-energy chemical space with fast quantum chemical methods. *Physical chemistry chemical physics : PCCP* **2020,** *22*, 7169-7192.

2. Frisch, M. J.; Trucks, G. W.; Schlegel, H. B.; Scuseria, G. E.; Robb, M. A.; Cheeseman, J. R.; Scalmani, G.; Barone, V.; Petersson, G. A.; Nakatsuji, H.; Li, X.; Caricato, M.; Marenich, A. V.; Bloino, J.; Janesko, B. G.; Gomperts, R.; Mennucci, B.; Hratchian, H. P.; Ortiz, J. V.; Izmaylov, A. F.; Sonnenberg, J. L.; Williams; Ding, F.; Lipparini, F.; Egidi, F.; Goings, J.; Peng, B.; Petrone, A.; Henderson, T.; Ranasinghe, D.; Zakrzewski, V. G.; Gao, J.; Rega, N.; Zheng, G.; Liang, W.; Hada, M.; Ehara, M.; Toyota, K.; Fukuda, R.; Hasegawa, J.; Ishida, M.; Nakajima, T.; Honda, Y.; Kitao, O.; Nakai, H.; Vreven, T.; Throssell, K.; Montgomery Jr., J. A.; Peralta, J. E.; Ogliaro, F.; Bearpark, M. J.; Heyd, J. J.; Brothers, E. N.; Kudin, K. N.; Staroverov, V. N.; Keith, T. A.; Kobayashi, R.; Normand, J.; Raghavachari, K.; Rendell, A. P.; Burant, J. C.; Iyengar, S. S.; Tomasi, J.; Cossi, M.; Millam, J. M.; Klene, M.; Adamo, C.; Cammi, R.; Ochterski, J. W.; Martin, R. L.; Morokuma, K.; Farkas, O.; Foresman, J. B.; Fox, D. J. *Gaussian 16 Rev. C.01*: Wallingford, CT, 2016.

3. Pescitelli, G.; Bruhn, T., Good Computational Practice in the Assignment of Absolute Configurations by TDDFT Calculations of ECD Spectra. *Chirality* **2016,** *28*, 466-74.

4. Lu, T.; Chen, F., Multiwfn: a multifunctional wavefunction analyzer. *J Comput Chem* **2012,** *33*, 580-92.

**Table S2.** Conformational analysis of the B3LYP/6-31G(d) optimized conformers of **1** in the gas phase (T=298.15 K)

| **Conformer** | **E*^a^* (Hartree)** | **C*^b^* (Hartree)** | **G*^c^* (kcal/mol)** | **ΔG*^d^* (kcal/mol)** | **Population*^e^*** |
| --- | --- | --- | --- | --- | --- |
| **1-1** | -766.5321896 | 0.19887 | -480874.1581 | 0 | 32.0% |
| **1-2** | -766.5321375 | 0.198891 | -480874.1122 | 0.045886565 | 29.6% |
| **1-3** | -766.5312419 | 0.198964 | -480873.5044 | 0.653703773 | 10.6% |
| **1-4** | -766.5309177 | 0.198962 | -480873.3022 | 0.85589996 | 7.5% |
| **1-5** | -766.5309604 | 0.199103 | -480873.2405 | 0.91756815 | 6.8% |
| **1-6** | -766.5304602 | 0.198856 | -480873.0816 | 1.07644864 | 5.2% |
| **1-7** | -766.5304551 | 0.198874 | -480873.0671 | 1.09095393 | 5.1% |
| **1-8** | -766.5301421 | 0.199017 | -480872.781 | 1.377060673 | 3.1% |

*^a^*Electronic energy obtained at M062X/6-311+G(2d,p) level of theory; *^b^*Thermal correction to Gibbs free energy obtained at B3LYP/6-31G(d) level of theory; *^c^*Gibbs free energy (E + C); *^d^*The relative Gibbs free energy; *^e^*The Boltzmann distribution of each conformer.

**Table S3.** Key transitions, oscillator strengths, and rotatory strengths in the ECD spectrum of conformer **1-1** at the B3LYP/6-31G(d) level of theory in MeOH with IEFPCM solvent model.

| ***Num^a^*** | ***Transition^b^*** | ***CI-coeff^b^*** | ***ΔE (eV)^d^*** | ***λ (nm)^e^*** | ***f^f^*** | ***R_vel_^g^*** | ***R_len_^h^*** |
| --- | --- | --- | --- | --- | --- | --- | --- |
| 1 | 58->60 | 0.64428 | 4.0591 | 305.45 | 0.0001 | -3.6521 | -1.3765 |
| 2 | 59->60 | 0.68105 | 4.3708 | 283.66 | 0.0802 | 16.4335 | 14.1442 |
| 3 | 57->60 | 0.61402 | 5.3566 | 231.46 | 0.0946 | 6.1286 | 6.2971 |
|  | 59->61 | -0.30399 |  |  |  |  |  |
| 4 | 57->60 | 0.32231 | 6.0986 | 203.30 | 0.8089 | -33.636 | -33.5406 |
|  | 59->61 | 0.60757 |  |  |  |  |  |
| 5 | 55->60 | -0.4246 | 6.4010 | 193.69 | 0.0098 | 6.0317 | 5.2692 |
|  | 56->60 | 0.46388 |  |  |  |  |  |
| 6 | 54->60 | -0.31876 | 6.5057 | 190.58 | 0.0535 | 0.2287 | -0.6527 |
|  | 55->60 | -0.28879 |  |  |  |  |  |
|  | 57->61 | 0.43091 |  |  |  |  |  |
|  | 59->62 | 0.22834 |  |  |  |  |  |
| 7 | 55->60 | 0.32797 | 6.6683 | 185.93 | 0.0761 | -22.0959 | -23.254 |
|  | 56->60 | 0.44787 |  |  |  |  |  |
|  | 57->61 | 0.27018 |  |  |  |  |  |
| 8 | 53->60 | 0.22865 | 6.8674 | 180.54 | 0.0419 | -2.2142 | -2.9029 |
|  | 54->60 | 0.41354 |  |  |  |  |  |
| 9 | 58->61 | 0.65753 | 6.9354 | 178.77 | 0.0007 | -4.4965 | -3.6414 |
| 10 | 55->61 | -0.33884 | 7.2534 | 170.93 | 0.1020 | -11.9883 | -11.9267 |
|  | 56->61 | 0.32725 |  |  |  |  |  |
|  | 57->61 | -0.25119 |  |  |  |  |  |
|  | 59->62 | 0.38958 |  |  |  |  |  |
| 11 | 52->60 | 0.43428 | 7.3654 | 168.33 | 0.0387 | -11.5796 | -12.8648 |
|  | 54->60 | 0.24578 |  |  |  |  |  |
|  | 58->62 | 0.28527 |  |  |  |  |  |
| 12 | 55->61 | 0.28736 | 7.4219 | 167.05 | 0.1129 | 19.8065 | 22.9776 |
|  | 56->61 | -0.27702 |  |  |  |  |  |
|  | 59->62 | 0.45149 |  |  |  |  |  |
| 13 | 51->60 | -0.26451 | 7.5438 | 164.35 | 0.1087 | 60.3232 | 64.5514 |
|  | 53->60 | 0.38025 |  |  |  |  |  |
|  | 54->60 | -0.30095 |  |  |  |  |  |
| 14 | 52->60 | -0.37316 | 7.7357 | 160.28 | 0.0075 | -29.1287 | -27.6332 |
|  | 58->62 | 0.48296 |  |  |  |  |  |
| 15 | 49->60 | 0.35251 | 7.9314 | 156.32 | 0.0154 | 18.1871 | 18.1418 |
|  | 50->60 | 0.28318 |  |  |  |  |  |
|  | 51->60 | 0.23341 |  |  |  |  |  |
|  | 54->61 | 0.28342 |  |  |  |  |  |
| 16 | 49->60 | -0.23223 | 7.9511 | 155.93 | 0.0596 | -3.4425 | -3.7119 |
|  | 50->60 | 0.22474 |  |  |  |  |  |
|  | 51->60 | 0.49123 |  |  |  |  |  |
|  | 53->60 | 0.28254 |  |  |  |  |  |
| 17 | 49->60 | -0.22923 | 8.0910 | 153.24 | 0.0897 | -5.8495 | -5.4221 |
|  | 54->61 | 0.22591 |  |  |  |  |  |
|  | 57->62 | 0.53916 |  |  |  |  |  |
| 18 | 59->64 | 0.48555 | 8.1030 | 153.01 | 0.0095 | -7.1545 | -1.9261 |
| 19 | 57->62 | 0.30823 | 8.1830 | 151.51 | 0.0415 | 7.6515 | 6.5848 |
| 20 | 59->63 | 0.45435 | 8.2250 | 150.74 | 0.0050 | 12.2182 | 14.3444 |
| 21 | 55->61 | 0.31349 | 8.3272 | 148.89 | 0.0157 | -22.6457 | -20.3128 |
|  | 55->65 | 0.23524 |  |  |  |  |  |
|  | 56->61 | 0.23479 |  |  |  |  |  |
|  | 56->65 | 0.23743 |  |  |  |  |  |
| 22 | 49->60 | -0.33326 | 8.3873 | 147.82 | 0.0266 | -12.4387 | -10.0448 |
|  | 50->60 | 0.37257 |  |  |  |  |  |
| 23 | 59->63 | -0.3144 | 8.4107 | 147.41 | 0.0197 | 12.8535 | 13.7269 |
|  | 59->66 | 0.39235 |  |  |  |  |  |
| 24 | 55->61 | 0.30987 | 8.4833 | 146.15 | 0.0048 | 4.3964 | 12.7068 |
|  | 56->61 | 0.24723 |  |  |  |  |  |
| 25 | 52->61 | 0.5552 | 8.5494 | 145.02 | 0.0031 | 2.4776 | 1.457 |
| 26 | 59->64 | 0.31459 | 8.6970 | 142.56 | 0.0237 | -10.7114 | -14.4702 |
|  | 59->66 | 0.32286 |  |  |  |  |  |
|  | 59->68 | 0.22605 |  |  |  |  |  |
| 27 | 46->60 | -0.27661 | 8.7645 | 141.46 | 0.0008 | -3.7755 | -1.8269 |
|  | 59->65 | 0.32435 |  |  |  |  |  |
| 28 | 59->65 | 0.42314 | 8.7768 | 141.26 | 0.0051 | -3.7984 | -6.5904 |
| 29 | 48->60 | 0.4464 | 8.8202 | 140.57 | 0.0056 | 3.9731 | 3.4334 |
| 30 | 51->61 | 0.34756 | 8.8945 | 139.39 | 0.0209 | 5.3631 | 4.8526 |
|  | 59->67 | -0.30235 |  |  |  |  |  |

*^a^*Number of the excited states; *^b^*Only transitions with contribution over 10.0% were listed; *^c^*Configuration-interaction coefficient; *^d^*Excitation energy; *^e^*Wavelength; *^f^*Oscillator strength; *^g^*Rotatory strength in velocity form (10^-40^ cgs); *^h^*Rotatory strength in length form (10^-40^ cgs).

**Table S4.** Key transitions, oscillator strengths, and rotatory strengths in the ECD spectrum of conformer **1-2** at the B3LYP/6-31G(d) level of theory in MeOH with IEFPCM solvent model.

| ***Num^a^*** | ***Transition^b^*** | ***CI-coeff^b^*** | ***ΔE (eV)^d^*** | ***λ (nm)^e^*** | ***f^f^*** | ***R_vel_^g^*** | ***R_len_^h^*** |
| --- | --- | --- | --- | --- | --- | --- | --- |
| 1 | 57->60 | 0.34927 | 4.0614 | 305.27 | 0.0001 | -2.9186 | -0.3296 |
|  | 58->60 | 0.54251 |  |  |  |  |  |
| 2 | 59->60 | 0.68129 | 4.3730 | 283.52 | 0.0807 | 16.9749 | 14.7808 |
| 3 | 57->60 | 0.51565 | 5.3570 | 231.44 | 0.0935 | 6.3316 | 6.2589 |
|  | 58->60 | -0.33153 |  |  |  |  |  |
|  | 59->61 | -0.30499 |  |  |  |  |  |
| 4 | 57->60 | 0.2693 | 6.0976 | 203.33 | 0.8050 | -23.3329 | -25.401 |
|  | 59->61 | 0.60675 |  |  |  |  |  |
| 5 | 55->60 | 0.47006 | 6.3699 | 194.64 | 0.0075 | -0.6096 | -1.1535 |
|  | 56->60 | -0.35954 |  |  |  |  |  |
| 6 | 54->60 | -0.23026 | 6.5409 | 189.55 | 0.0804 | 4.8087 | 6.7687 |
|  | 56->60 | -0.28249 |  |  |  |  |  |
|  | 57->61 | 0.40622 |  |  |  |  |  |
|  | 58->61 | -0.25813 |  |  |  |  |  |
|  | 59->62 | 0.2535 |  |  |  |  |  |
| 7 | 55->60 | 0.35739 | 6.6704 | 185.87 | 0.0511 | -17.4452 | -18.6598 |
|  | 56->60 | 0.49434 |  |  |  |  |  |
| 8 | 53->60 | 0.24317 | 6.8776 | 180.27 | 0.0427 | 7.7991 | 8.1836 |
|  | 54->60 | 0.41805 |  |  |  |  |  |
|  | 58->61 | -0.27807 |  |  |  |  |  |
| 9 | 57->61 | 0.36118 | 6.9528 | 178.32 | 0.0002 | 1.746 | 0.6489 |
|  | 58->61 | 0.5032 |  |  |  |  |  |
| 10 | 55->61 | 0.35367 | 7.2319 | 171.44 | 0.1023 | 23.0471 | 20.6833 |
|  | 56->61 | -0.34624 |  |  |  |  |  |
|  | 57->61 | -0.26564 |  |  |  |  |  |
|  | 59->62 | 0.34584 |  |  |  |  |  |
| 11 | 51->60 | -0.25488 | 7.3416 | 168.88 | 0.0310 | 17.6155 | 19.4687 |
|  | 52->60 | 0.34797 |  |  |  |  |  |
|  | 53->60 | 0.25891 |  |  |  |  |  |
|  | 54->60 | -0.34035 |  |  |  |  |  |
| 12 | 55->61 | -0.25729 | 7.4099 | 167.32 | 0.1563 | 7.7379 | 5.8945 |
|  | 56->61 | 0.28365 |  |  |  |  |  |
|  | 59->62 | 0.47152 |  |  |  |  |  |
| 13 | 51->60 | 0.25956 | 7.5320 | 164.61 | 0.0563 | -39.8801 | -43.0367 |
|  | 52->60 | 0.27218 |  |  |  |  |  |
|  | 54->60 | 0.22517 |  |  |  |  |  |
|  | 58->62 | 0.31709 |  |  |  |  |  |
| 14 | 52->60 | 0.39557 | 7.7349 | 160.29 | 0.0197 | 44.8316 | 46.4152 |
|  | 57->62 | -0.25056 |  |  |  |  |  |
|  | 58->62 | -0.32485 |  |  |  |  |  |
| 15 | 49->60 | -0.2581 | 7.8918 | 157.11 | 0.0525 | -26.8862 | -28.9913 |
|  | 51->60 | 0.3503 |  |  |  |  |  |
|  | 53->60 | 0.363 |  |  |  |  |  |
| 16 | 51->60 | 0.31119 | 8.0221 | 154.55 | 0.0115 | -0.3142 | 0.4721 |
|  | 54->61 | 0.2602 |  |  |  |  |  |
| 17 | 49->60 | -0.31337 | 8.0958 | 153.15 | 0.1055 | 31.3484 | 30.7203 |
|  | 57->62 | 0.3863 |  |  |  |  |  |
|  | 58->62 | -0.24374 |  |  |  |  |  |
| 18 | 57->62 | 0.29003 | 8.1203 | 152.68 | 0.0464 | -10.8168 | -11.097 |
|  | 59->63 | -0.31749 |  |  |  |  |  |
|  | 59->64 | 0.35938 |  |  |  |  |  |
| 19 | 49->60 | 0.25396 | 8.1925 | 151.34 | 0.0128 | 3.5244 | 2.4355 |
|  | 50->60 | 0.29623 |  |  |  |  |  |
| 20 | 56->64 | 0.23678 | 8.2450 | 150.38 | 0.0034 | 3.5085 | -4.8349 |
|  | 59->63 | 0.37386 |  |  |  |  |  |
|  | 59->66 | 0.25729 |  |  |  |  |  |
| 21 | 54->61 | -0.25785 | 8.3277 | 148.88 | 0.0095 | -19.464 | -13.1408 |
|  | 55->61 | 0.28379 |  |  |  |  |  |
|  | 56->61 | 0.24307 |  |  |  |  |  |
| 22 | 49->60 | -0.32942 | 8.3792 | 147.97 | 0.0318 | -20.6509 | -21.0249 |
|  | 50->60 | 0.39007 |  |  |  |  |  |
| 23 | 59->63 | -0.33102 | 8.4073 | 147.47 | 0.0151 | 4.8461 | 4.7576 |
|  | 59->66 | 0.41117 |  |  |  |  |  |
| 24 | 52->61 | 0.23489 | 8.4625 | 146.51 | 0.0054 | 9.0828 | 11.3791 |
|  | 54->61 | -0.25097 |  |  |  |  |  |
|  | 56->61 | 0.26776 |  |  |  |  |  |
| 25 | 52->61 | 0.40843 | 8.5783 | 144.53 | 0.0024 | -11.4075 | -6.7649 |
|  | 55->61 | -0.28505 |  |  |  |  |  |
| 26 | 59->64 | 0.36023 | 8.6979 | 142.54 | 0.0056 | 21.9346 | 21.585 |
| 27 | 45->60 | 0.22488 | 8.7364 | 141.92 | 0.0057 | 0.7155 | 2.7519 |
|  | 46->60 | -0.28042 |  |  |  |  |  |
|  | 47->60 | 0.33009 |  |  |  |  |  |
| 28 | 59->65 | 0.39325 | 8.7913 | 141.03 | 0.0071 | -2.8361 | 0.5393 |
|  | 59->67 | -0.22802 |  |  |  |  |  |
|  | 59->68 | 0.23388 |  |  |  |  |  |
| 29 | 51->61 | 0.29496 | 8.8147 | 140.66 | 0.0141 | 16.2683 | 12.0583 |
|  | 52->61 | 0.2786 |  |  |  |  |  |
|  | 59->65 | 0.25017 |  |  |  |  |  |
| 30 | 43->60 | -0.2413 | 8.8530 | 140.05 | 0.0126 | 4.3223 | 0.7338 |
|  | 48->60 | 0.41998 |  |  |  |  |  |

*^a^*Number of the excited states; *^b^*Only transitions with contribution over 10.0% were listed; *^c^*Configuration-interaction coefficient; *^d^*Excitation energy; *^e^*Wavelength; *^f^*Oscillator strength; *^g^*Rotatory strength in velocity form (10^-40^ cgs); *^h^*Rotatory strength in length form (10^-40^ cgs).

**Table S5.** Key transitions, oscillator strengths, and rotatory strengths in the ECD spectrum of conformer **1-3** at the B3LYP/6-31G(d) level of theory in MeOH with IEFPCM solvent model.

| ***Num^a^*** | ***Transition^b^*** | ***CI-coeff^b^*** | ***ΔE (eV)^d^*** | ***λ (nm)^e^*** | ***f^f^*** | ***R_vel_^g^*** | ***R_len_^h^*** |
| --- | --- | --- | --- | --- | --- | --- | --- |
| 1 | 58->60 | 0.61975 | 4.0564 | 305.65 | 0.0002 | 1.9571 | 4.6014 |
| 2 | 59->60 | 0.68272 | 4.3537 | 284.78 | 0.0807 | 11.1294 | 8.7592 |
| 3 | 57->60 | 0.59215 | 5.3663 | 231.04 | 0.0938 | 5.8965 | 5.739 |
|  | 59->61 | 0.30828 |  |  |  |  |  |
| 4 | 57->60 | -0.3099 | 6.0922 | 203.51 | 0.7812 | -44.2436 | -44.8753 |
|  | 59->61 | 0.60799 |  |  |  |  |  |
| 5 | 55->60 | 0.58898 | 6.4526 | 192.15 | 0.0004 | 5.9811 | 5.2223 |
|  | 56->60 | -0.322 |  |  |  |  |  |
| 6 | 54->60 | 0.3137 | 6.4938 | 190.93 | 0.0557 | 5.6368 | 5.492 |
|  | 57->61 | 0.45839 |  |  |  |  |  |
|  | 59->62 | -0.27552 |  |  |  |  |  |
| 7 | 53->60 | -0.24425 | 6.6651 | 186.02 | 0.0637 | -23.4949 | -24.847 |
|  | 55->60 | 0.24413 |  |  |  |  |  |
|  | 56->60 | 0.50269 |  |  |  |  |  |
| 8 | 53->60 | 0.36759 | 6.8727 | 180.40 | 0.0419 | -0.7246 | -1.159 |
|  | 54->60 | -0.35925 |  |  |  |  |  |
|  | 56->60 | 0.26974 |  |  |  |  |  |
| 9 | 58->61 | 0.63434 | 6.9541 | 178.29 | 0.0059 | 11.3148 | 12.1897 |
| 10 | 53->60 | 0.39219 | 7.2606 | 170.76 | 0.0373 | 11.672 | 11.7082 |
|  | 54->60 | 0.43592 |  |  |  |  |  |
| 11 | 55->61 | 0.30674 | 7.3413 | 168.89 | 0.2301 | 1.0401 | 3.8103 |
|  | 57->61 | 0.32167 |  |  |  |  |  |
|  | 59->62 | 0.48074 |  |  |  |  |  |
| 12 | 55->61 | 0.43534 | 7.4306 | 166.86 | 0.0905 | 23.1501 | 20.8945 |
|  | 56->61 | -0.25726 |  |  |  |  |  |
|  | 59->62 | -0.3426 |  |  |  |  |  |
| 13 | 58->62 | 0.4871 | 7.5960 | 163.22 | 0.0065 | -24.6788 | -23.1381 |
| 14 | 50->60 | -0.24952 | 7.7734 | 159.50 | 0.0682 | 15.1559 | 15.9474 |
|  | 52->60 | 0.54436 |  |  |  |  |  |
| 15 | 49->60 | 0.31411 | 7.9306 | 156.34 | 0.0051 | 16.4305 | 17.0147 |
|  | 50->60 | 0.32095 |  |  |  |  |  |
|  | 53->61 | -0.3 |  |  |  |  |  |
|  | 54->61 | 0.23899 |  |  |  |  |  |
| 16 | 51->60 | 0.4799 | 7.9826 | 155.32 | 0.0660 | -42.5385 | -43.1783 |
|  | 59->64 | 0.22978 |  |  |  |  |  |
| 17 | 51->60 | -0.25107 | 8.0348 | 154.31 | 0.0123 | -2.5431 | 3.0009 |
|  | 59->64 | 0.46479 |  |  |  |  |  |
| 18 | 54->61 | -0.25179 | 8.1156 | 152.77 | 0.0665 | 13.5223 | 14.9033 |
|  | 57->62 | 0.42396 |  |  |  |  |  |
| 19 | 53->61 | -0.28391 | 8.1602 | 151.94 | 0.0316 | 10.2543 | 11.1399 |
|  | 56->61 | 0.25247 |  |  |  |  |  |
|  | 57->62 | 0.35245 |  |  |  |  |  |
| 20 | 55->64 | -0.23247 | 8.1978 | 151.24 | 0.0139 | 22.26 | 13.0944 |
|  | 59->63 | 0.37986 |  |  |  |  |  |
|  | 59->66 | -0.37451 |  |  |  |  |  |
| 21 | 59->63 | 0.41767 | 8.3200 | 149.02 | 0.0042 | -7.8576 | -6.7169 |
|  | 59->65 | -0.37793 |  |  |  |  |  |
| 22 | 56->61 | 0.31955 | 8.3340 | 148.77 | 0.0088 | -25.3472 | -21.2847 |
|  | 56->63 | -0.23662 |  |  |  |  |  |
|  | 56->65 | 0.25211 |  |  |  |  |  |
| 23 | 48->60 | 0.41182 | 8.4123 | 147.38 | 0.0038 | 4.0046 | 4.6475 |
|  | 54->61 | -0.28935 |  |  |  |  |  |
| 24 | 48->60 | 0.3454 | 8.4486 | 146.75 | 0.0094 | 36.1151 | 39.3185 |
|  | 50->60 | -0.23129 |  |  |  |  |  |
|  | 54->61 | 0.23681 |  |  |  |  |  |
| 25 | 55->61 | 0.22488 | 8.4948 | 145.95 | 0.0214 | -27.4972 | -23.5404 |
|  | 56->61 | 0.27887 |  |  |  |  |  |
| 26 | 49->60 | 0.35237 | 8.5288 | 145.37 | 0.0288 | 0.5647 | 0.6347 |
|  | 50->60 | -0.28476 |  |  |  |  |  |
| 27 | 55->64 | 0.24812 | 8.7068 | 142.40 | 0.0313 | -20.9851 | -24.638 |
|  | 59->64 | 0.33787 |  |  |  |  |  |
|  | 59->66 | -0.24096 |  |  |  |  |  |
| 28 | 59->65 | 0.39648 | 8.7512 | 141.68 | 0.0015 | 2.4965 | 4.5334 |
|  | 59->66 | 0.34643 |  |  |  |  |  |
|  | 59->67 | 0.33109 |  |  |  |  |  |
| 29 | 43->60 | -0.24133 | 8.9326 | 138.80 | 0.0142 | -51.8765 | -51.0147 |
|  | 58->63 | 0.42647 |  |  |  |  |  |
| 30 | 43->60 | 0.28094 | 8.9405 | 138.68 | 0.0110 | 28.3565 | 28.1271 |
|  | 58->63 | 0.36786 |  |  |  |  |  |

*^a^*Number of the excited states; *^b^*Only transitions with contribution over 10.0% were listed; *^c^*Configuration-interaction coefficient; *^d^*Excitation energy; *^e^*Wavelength; *^f^*Oscillator strength; *^g^*Rotatory strength in velocity form (10^-40^ cgs); *^h^*Rotatory strength in length form (10^-40^ cgs).

**Table S6.** Key transitions, oscillator strengths, and rotatory strengths in the ECD spectrum of conformer **1-4** at the B3LYP/6-31G(d) level of theory in MeOH with IEFPCM solvent model.

| ***Num^a^*** | ***Transition^b^*** | ***CI-coeff^b^*** | ***ΔE (eV)^d^*** | ***λ (nm)^e^*** | ***f^f^*** | ***R_vel_^g^*** | ***R_len_^h^*** |
| --- | --- | --- | --- | --- | --- | --- | --- |
| 1 | 57->60 | 0.29216 | 4.0622 | 305.21 | 0.0002 | 2.6898 | 5.3061 |
|  | 58->60 | 0.56825 |  |  |  |  |  |
| 2 | 59->60 | 0.68178 | 4.3592 | 284.42 | 0.0833 | 11.0324 | 8.9509 |
| 3 | 57->60 | 0.54418 | 5.3519 | 231.66 | 0.0964 | 7.2414 | 6.8287 |
|  | 58->60 | -0.28477 |  |  |  |  |  |
|  | 59->61 | -0.30145 |  |  |  |  |  |
| 4 | 57->60 | 0.28364 | 6.0919 | 203.52 | 0.7998 | -33.4305 | -36.3343 |
|  | 59->61 | 0.60829 |  |  |  |  |  |
| 5 | 55->60 | -0.39815 | 6.3598 | 194.95 | 0.0084 | 0.2596 | -0.4959 |
|  | 56->60 | 0.45224 |  |  |  |  |  |
| 6 | 55->60 | 0.24438 | 6.5329 | 189.78 | 0.0890 | 0.9647 | 2.9413 |
|  | 57->61 | 0.42921 |  |  |  |  |  |
|  | 58->61 | -0.22746 |  |  |  |  |  |
|  | 59->62 | -0.27801 |  |  |  |  |  |
| 7 | 55->60 | 0.34632 | 6.7416 | 183.91 | 0.0091 | -6.996 | -7.3595 |
|  | 56->60 | 0.47196 |  |  |  |  |  |
| 8 | 51->60 | -0.25679 | 6.8586 | 180.77 | 0.0560 | 3.7496 | 4.1601 |
|  | 54->60 | 0.45286 |  |  |  |  |  |
| 9 | 57->61 | 0.30678 | 6.9640 | 178.04 | 0.0010 | 4.4675 | 3.6967 |
|  | 58->61 | 0.56273 |  |  |  |  |  |
| 10 | 55->61 | -0.33046 | 7.2147 | 171.85 | 0.1221 | 17.9202 | 16.0994 |
|  | 56->61 | 0.32117 |  |  |  |  |  |
|  | 57->61 | 0.29663 |  |  |  |  |  |
|  | 59->62 | 0.37375 |  |  |  |  |  |
| 11 | 51->60 | 0.27201 | 7.3799 | 168.00 | 0.0475 | 0.2549 | -1.2756 |
|  | 53->60 | 0.38356 |  |  |  |  |  |
|  | 54->60 | 0.25135 |  |  |  |  |  |
|  | 59->62 | 0.26095 |  |  |  |  |  |
| 12 | 53->60 | -0.24282 | 7.3886 | 167.81 | 0.1034 | 11.3153 | 11.0261 |
|  | 55->61 | 0.23231 |  |  |  |  |  |
|  | 56->61 | -0.27106 |  |  |  |  |  |
|  | 59->62 | 0.36606 |  |  |  |  |  |
| 13 | 51->60 | 0.35041 | 7.4939 | 165.45 | 0.0730 | -35.3598 | -38.5074 |
|  | 54->60 | 0.25297 |  |  |  |  |  |
|  | 58->62 | 0.29088 |  |  |  |  |  |
| 14 | 51->60 | -0.22901 | 7.7358 | 160.27 | 0.0152 | 37.1283 | 38.0285 |
|  | 52->60 | 0.24868 |  |  |  |  |  |
|  | 53->60 | 0.23102 |  |  |  |  |  |
|  | 57->62 | 0.24578 |  |  |  |  |  |
|  | 58->62 | 0.379 |  |  |  |  |  |
| 15 | 50->60 | 0.35311 | 7.9675 | 155.61 | 0.0339 | -13.8765 | -13.7483 |
|  | 52->60 | 0.47454 |  |  |  |  |  |
| 16 | 50->60 | 0.30979 | 8.0251 | 154.49 | 0.0394 | 0.3355 | 0.3993 |
|  | 51->61 | -0.22709 |  |  |  |  |  |
|  | 54->61 | 0.31009 |  |  |  |  |  |
| 17 | 50->60 | -0.25545 | 8.0591 | 153.84 | 0.0306 | 13.9129 | 11.9228 |
|  | 57->62 | 0.41186 |  |  |  |  |  |
|  | 58->62 | -0.26929 |  |  |  |  |  |
| 18 | 57->62 | 0.28586 | 8.1022 | 153.03 | 0.0594 | -29.1927 | -33.2627 |
|  | 59->63 | 0.43538 |  |  |  |  |  |
|  | 59->64 | -0.23593 |  |  |  |  |  |
| 19 | 50->60 | 0.36402 | 8.1858 | 151.46 | 0.1025 | 4.8427 | 6.5994 |
|  | 52->60 | -0.24436 |  |  |  |  |  |
|  | 57->62 | 0.23168 |  |  |  |  |  |
| 20 | 59->63 | 0.26846 | 8.2109 | 151.00 | 0.0072 | 25.6963 | 20.7264 |
|  | 59->64 | 0.35986 |  |  |  |  |  |
| 21 | 48->60 | 0.2437 | 8.3214 | 148.99 | 0.0030 | -15.7109 | -19.1108 |
|  | 49->60 | 0.32458 |  |  |  |  |  |
| 22 | 59->66 | 0.25477 | 8.3351 | 148.75 | 0.0124 | 19.4587 | 13.8049 |
| 23 | 54->61 | 0.24999 | 8.3671 | 148.18 | 0.0183 | -9.7274 | -10.3463 |
|  | 55->61 | 0.28182 |  |  |  |  |  |
|  | 56->61 | 0.27671 |  |  |  |  |  |
|  | 59->66 | 0.25697 |  |  |  |  |  |
| 24 | 55->61 | 0.25827 | 8.3868 | 147.83 | 0.0098 | 20.9279 | 17.6981 |
|  | 56->61 | 0.30275 |  |  |  |  |  |
| 25 | 52->61 | 0.23194 | 8.5835 | 144.45 | 0.0040 | -5.0726 | -2.5615 |
|  | 53->61 | 0.3997 |  |  |  |  |  |
|  | 55->61 | -0.2615 |  |  |  |  |  |
| 26 | 59->64 | 0.26501 | 8.6749 | 142.92 | 0.0024 | 16.2669 | 13.1345 |
|  | 59->65 | 0.34716 |  |  |  |  |  |
| 27 | 46->60 | -0.23578 | 8.6835 | 142.78 | 0.0105 | 5.5668 | 6.9864 |
|  | 47->60 | 0.29884 |  |  |  |  |  |
|  | 59->65 | -0.26817 |  |  |  |  |  |
| 28 | 59->64 | -0.27664 | 8.7219 | 142.15 | 0.0067 | 2.8981 | 7.4595 |
|  | 59->66 | 0.31871 |  |  |  |  |  |
| 29 | 51->61 | -0.30175 | 8.8562 | 140.00 | 0.0300 | 38.7141 | 35.4402 |
|  | 52->61 | 0.32466 |  |  |  |  |  |
| 30 | 57->63 | 0.24459 | 8.8922 | 139.43 | 0.0112 | 13.3728 | 10.7507 |
|  | 58->63 | 0.48091 |  |  |  |  |  |
|  | 58->65 | 0.2258 |  |  |  |  |  |

*^a^*Number of the excited states; *^b^*Only transitions with contribution over 10.0% were listed; *^c^*Configuration-interaction coefficient; *^d^*Excitation energy; *^e^*Wavelength; *^f^*Oscillator strength; *^g^*Rotatory strength in velocity form (10^-40^ cgs); *^h^*Rotatory strength in length form (10^-40^ cgs).

**Table S7.** Key transitions, oscillator strengths, and rotatory strengths in the ECD spectrum of conformer **1-5** at the B3LYP/6-31G(d) level of theory in MeOH with IEFPCM solvent model.

| ***Num^a^*** | ***Transition^b^*** | ***CI-coeff^b^*** | ***ΔE (eV)^d^*** | ***λ (nm)^e^*** | ***f^f^*** | ***R_vel_^g^*** | ***R_len_^h^*** |
| --- | --- | --- | --- | --- | --- | --- | --- |
| 1 | 58->60 | 0.63185 | 4.0611 | 305.30 | 0.0002 | 2.0513 | 4.3988 |
| 2 | 59->60 | 0.68207 | 4.3555 | 284.66 | 0.0829 | 10.4128 | 8.1859 |
| 3 | 57->60 | 0.61158 | 5.3514 | 231.69 | 0.0973 | 6.6054 | 6.435 |
|  | 59->61 | -0.30073 |  |  |  |  |  |
| 4 | 57->60 | 0.31621 | 6.0917 | 203.53 | 0.8048 | -42.9473 | -43.9277 |
|  | 59->61 | 0.60918 |  |  |  |  |  |
| 5 | 55->60 | 0.53764 | 6.3831 | 194.24 | 0.0118 | 5.1098 | 4.0175 |
|  | 56->60 | -0.31724 |  |  |  |  |  |
| 6 | 54->60 | 0.31629 | 6.5154 | 190.29 | 0.0720 | -1.1923 | -1.7626 |
|  | 57->61 | 0.47263 |  |  |  |  |  |
|  | 59->62 | -0.27053 |  |  |  |  |  |
| 7 | 54->60 | -0.29542 | 6.7393 | 183.97 | 0.0222 | -11.5096 | -12.0899 |
|  | 55->60 | 0.30729 |  |  |  |  |  |
|  | 56->60 | 0.4324 |  |  |  |  |  |
| 8 | 54->60 | 0.39217 | 6.8472 | 181.07 | 0.0545 | -5.6848 | -6.0562 |
|  | 56->60 | 0.32896 |  |  |  |  |  |
| 9 | 58->61 | 0.65124 | 6.9470 | 178.47 | 0.0017 | -4.5814 | -3.3992 |
| 10 | 55->61 | -0.37441 | 7.2417 | 171.21 | 0.1306 | -16.2207 | -16.5719 |
|  | 57->61 | 0.2986 |  |  |  |  |  |
|  | 59->62 | 0.42507 |  |  |  |  |  |
| 11 | 52->60 | -0.24314 | 7.3407 | 168.90 | 0.0406 | -10.0306 | -11.6613 |
|  | 53->60 | 0.34122 |  |  |  |  |  |
|  | 54->60 | -0.25048 |  |  |  |  |  |
|  | 58->62 | -0.28219 |  |  |  |  |  |
| 12 | 55->61 | 0.41204 | 7.4066 | 167.40 | 0.1101 | 21.1413 | 25.2567 |
|  | 59->62 | 0.40332 |  |  |  |  |  |
| 13 | 51->60 | 0.41972 | 7.6227 | 162.65 | 0.0718 | 51.7501 | 54.4852 |
|  | 52->60 | 0.24185 |  |  |  |  |  |
|  | 58->62 | 0.26538 |  |  |  |  |  |
| 14 | 51->60 | -0.23795 | 7.7252 | 160.49 | 0.0162 | -33.6514 | -32.9761 |
|  | 53->60 | 0.36665 |  |  |  |  |  |
|  | 58->62 | 0.39311 |  |  |  |  |  |
| 15 | 49->60 | 0.25649 | 7.8442 | 158.06 | 0.0266 | 22.0671 | 24.0648 |
|  | 50->60 | 0.34055 |  |  |  |  |  |
|  | 52->60 | 0.36651 |  |  |  |  |  |
| 16 | 50->60 | 0.27824 | 8.0340 | 154.33 | 0.0417 | -3.4343 | -5.343 |
|  | 51->61 | 0.24351 |  |  |  |  |  |
|  | 54->61 | 0.37847 |  |  |  |  |  |
| 17 | 57->62 | 0.57507 | 8.0686 | 153.66 | 0.0686 | -7.4763 | -7.5457 |
| 18 | 59->63 | -0.27447 | 8.0985 | 153.10 | 0.0066 | -22.9136 | -20.6103 |
|  | 59->64 | 0.44891 |  |  |  |  |  |
| 19 | 59->63 | 0.48966 | 8.1687 | 151.78 | 0.0174 | -1.593 | -2.6925 |
| 20 | 50->60 | 0.39128 | 8.2005 | 151.19 | 0.0980 | 34.3213 | 37.2047 |
|  | 52->60 | -0.23017 |  |  |  |  |  |
| 21 | 48->60 | 0.2398 | 8.3275 | 148.89 | 0.0027 | -17.8422 | -17.5661 |
|  | 49->60 | 0.33414 |  |  |  |  |  |
|  | 51->60 | 0.229 |  |  |  |  |  |
| 22 | 56->63 | 0.27086 | 8.3377 | 148.70 | 0.0130 | 26.2939 | 16.6337 |
|  | 56->65 | -0.25234 |  |  |  |  |  |
| 23 | 56->61 | 0.38485 | 8.3688 | 148.15 | 0.0298 | 5.1328 | 9.5465 |
|  | 59->66 | 0.22706 |  |  |  |  |  |
| 24 | 56->61 | 0.36199 | 8.3886 | 147.80 | 0.0043 | 13.1904 | 9.897 |
| 25 | 52->61 | -0.24658 | 8.5668 | 144.73 | 0.0026 | -0.7721 | 2.3221 |
|  | 53->61 | 0.45748 |  |  |  |  |  |
| 26 | 59->64 | 0.30714 | 8.6307 | 143.66 | 0.0130 | -17.9287 | -22.6902 |
|  | 59->65 | 0.38415 |  |  |  |  |  |
| 27 | 46->60 | 0.32328 | 8.7238 | 142.12 | 0.0073 | -3.4471 | -4.0632 |
|  | 47->60 | -0.28435 |  |  |  |  |  |
| 28 | 59->66 | 0.42546 | 8.8373 | 140.30 | 0.0097 | 27.7353 | 26.6662 |
|  | 59->67 | -0.22948 |  |  |  |  |  |
| 29 | 58->63 | 0.38439 | 8.8736 | 139.72 | 0.0165 | 17.5018 | 14.6121 |
|  | 59->67 | -0.32676 |  |  |  |  |  |
| 30 | 45->60 | 0.30294 | 8.9002 | 139.30 | 0.0162 | -17.2679 | -20.9656 |
|  | 46->60 | 0.397 |  |  |  |  |  |
|  | 48->60 | 0.23585 |  |  |  |  |  |

*^a^*Number of the excited states; *^b^*Only transitions with contribution over 10.0% were listed; *^c^*Configuration-interaction coefficient; *^d^*Excitation energy; *^e^*Wavelength; *^f^*Oscillator strength; *^g^*Rotatory strength in velocity form (10^-40^ cgs); *^h^*Rotatory strength in length form (10^-40^ cgs).

**Table S8.** Key transitions, oscillator strengths, and rotatory strengths in the ECD spectrum of conformer **1-6** at the B3LYP/6-31G(d) level of theory in MeOH with IEFPCM solvent model.

| ***Num^a^*** | ***Transition^b^*** | ***CI-coeff^b^*** | ***ΔE (eV)^d^*** | ***λ (nm)^e^*** | ***f^f^*** | ***R_vel_^g^*** | ***R_len_^h^*** |
| --- | --- | --- | --- | --- | --- | --- | --- |
| 1 | 57->60 | 0.54963 | 4.0563 | 305.66 | 0.0002 | -0.458 | -2.6571 |
|  | 58->60 | 0.35953 |  |  |  |  |  |
| 2 | 59->60 | 0.68225 | 4.3507 | 284.97 | 0.0777 | -9.4213 | -6.9404 |
| 3 | 57->60 | -0.33315 | 5.3340 | 232.44 | 0.0914 | -8.6923 | -8.1701 |
|  | 58->60 | 0.51311 |  |  |  |  |  |
|  | 59->61 | -0.30554 |  |  |  |  |  |
| 4 | 58->60 | 0.27195 | 6.0725 | 204.17 | 0.7789 | 20.8494 | 24.6796 |
|  | 59->61 | 0.60117 |  |  |  |  |  |
| 5 | 56->60 | 0.56837 | 6.3081 | 196.55 | 0.0151 | 1.5353 | 3.9513 |
| 6 | 55->60 | 0.5911 | 6.4393 | 192.54 | 0.0155 | -5.3524 | -6.7118 |
| 7 | 56->60 | 0.38387 | 6.6358 | 186.84 | 0.1077 | -8.8437 | -11.031 |
|  | 58->61 | -0.33665 |  |  |  |  |  |
|  | 59->62 | 0.25066 |  |  |  |  |  |
| 8 | 53->60 | 0.43315 | 6.8430 | 181.18 | 0.0605 | 3.1395 | 2.7262 |
|  | 54->60 | -0.3448 |  |  |  |  |  |
| 9 | 57->61 | 0.54391 | 6.9571 | 178.21 | 0.0012 | -3.3878 | -2.4378 |
|  | 58->61 | 0.35075 |  |  |  |  |  |
| 10 | 55->61 | 0.35767 | 7.2068 | 172.04 | 0.1309 | -11.7686 | -9.7021 |
|  | 56->61 | 0.2866 |  |  |  |  |  |
|  | 57->61 | -0.23188 |  |  |  |  |  |
|  | 59->62 | 0.38097 |  |  |  |  |  |
| 11 | 52->60 | 0.42967 | 7.3302 | 169.14 | 0.0128 | -8.4829 | -8.9261 |
|  | 54->60 | 0.3466 |  |  |  |  |  |
| 12 | 55->61 | -0.35189 | 7.3812 | 167.97 | 0.1603 | 4.8411 | 7.6836 |
|  | 56->61 | -0.22765 |  |  |  |  |  |
|  | 59->62 | 0.43885 |  |  |  |  |  |
| 13 | 49->60 | -0.22674 | 7.4822 | 165.71 | 0.0485 | 19.9404 | 21.1056 |
|  | 53->60 | 0.25049 |  |  |  |  |  |
|  | 54->60 | 0.36846 |  |  |  |  |  |
| 14 | 52->60 | 0.33424 | 7.7339 | 160.31 | 0.0058 | -24.0353 | -23.2475 |
|  | 53->60 | -0.27061 |  |  |  |  |  |
|  | 57->62 | 0.40121 |  |  |  |  |  |
|  | 58->62 | 0.23618 |  |  |  |  |  |
| 15 | 55->61 | -0.22592 | 7.8914 | 157.11 | 0.0163 | -3.6058 | -4.5854 |
|  | 56->61 | 0.34544 |  |  |  |  |  |
|  | 59->63 | -0.31377 |  |  |  |  |  |
| 16 | 49->60 | -0.35448 | 7.9082 | 156.78 | 0.0155 | 29.5816 | 32.1779 |
|  | 51->60 | 0.35786 |  |  |  |  |  |
|  | 53->60 | -0.23288 |  |  |  |  |  |
|  | 53->61 | 0.24937 |  |  |  |  |  |
| 17 | 51->60 | 0.44241 | 8.0146 | 154.70 | 0.0476 | 13.4883 | 13.8586 |
|  | 59->63 | 0.25737 |  |  |  |  |  |
| 18 | 51->60 | -0.26907 | 8.0331 | 154.34 | 0.0116 | -2.3328 | -2.5485 |
|  | 58->62 | 0.3273 |  |  |  |  |  |
|  | 59->63 | 0.30309 |  |  |  |  |  |
| 19 | 49->60 | 0.26446 | 8.1151 | 152.78 | 0.0590 | -16.7817 | -15.9413 |
|  | 55->61 | 0.22994 |  |  |  |  |  |
|  | 58->62 | 0.28273 |  |  |  |  |  |
|  | 59->64 | 0.28567 |  |  |  |  |  |
| 20 | 55->64 | 0.23061 | 8.1630 | 151.89 | 0.0463 | 26.4325 | 33.7878 |
|  | 56->61 | 0.26819 |  |  |  |  |  |
|  | 58->62 | -0.28756 |  |  |  |  |  |
|  | 59->64 | 0.38017 |  |  |  |  |  |
| 21 | 49->60 | 0.28903 | 8.2426 | 150.42 | 0.0154 | 6.1541 | 5.684 |
|  | 53->61 | 0.36037 |  |  |  |  |  |
|  | 54->61 | -0.31741 |  |  |  |  |  |
| 22 | 59->65 | -0.27089 | 8.3175 | 149.06 | 0.0063 | -12.6155 | -13.1587 |
|  | 59->66 | 0.46381 |  |  |  |  |  |
| 23 | 52->61 | -0.22864 | 8.4734 | 146.32 | 0.0056 | -7.3509 | -17.6842 |
|  | 56->63 | -0.24617 |  |  |  |  |  |
|  | 56->65 | 0.27001 |  |  |  |  |  |
| 24 | 47->60 | 0.35759 | 8.4848 | 146.12 | 0.0006 | 5.2829 | 5.4653 |
|  | 48->60 | -0.29822 |  |  |  |  |  |
|  | 50->60 | -0.30021 |  |  |  |  |  |
| 25 | 52->61 | 0.37194 | 8.5613 | 144.82 | 0.0361 | 22.5436 | 21.7346 |
|  | 59->63 | -0.23959 |  |  |  |  |  |
| 26 | 48->60 | -0.31473 | 8.5902 | 144.33 | 0.0624 | -27.7743 | -21.7082 |
|  | 50->60 | 0.4371 |  |  |  |  |  |
| 27 | 52->61 | 0.27274 | 8.6364 | 143.56 | 0.0010 | -5.9888 | -4.2138 |
|  | 59->65 | 0.42053 |  |  |  |  |  |
|  | 59->66 | 0.26348 |  |  |  |  |  |
| 28 | 55->64 | -0.24952 | 8.7067 | 142.40 | 0.0142 | 2.2947 | -3.5114 |
|  | 59->64 | 0.35461 |  |  |  |  |  |
|  | 59->66 | -0.26131 |  |  |  |  |  |
| 29 | 51->61 | 0.3574 | 8.8736 | 139.72 | 0.0379 | -52.2374 | -49.778 |
|  | 54->61 | 0.26037 |  |  |  |  |  |
| 30 | 57->63 | -0.24041 | 8.9146 | 139.08 | 0.0090 | -14.4774 | -14.7466 |
|  | 59->67 | 0.29294 |  |  |  |  |  |

*^a^*Number of the excited states; *^b^*Only transitions with contribution over 10.0% were listed; *^c^*Configuration-interaction coefficient; *^d^*Excitation energy; *^e^*Wavelength; *^f^*Oscillator strength; *^g^*Rotatory strength in velocity form (10^-40^ cgs); *^h^*Rotatory strength in length form (10^-40^ cgs).

**Table S9.** Key transitions, oscillator strengths, and rotatory strengths in the ECD spectrum of conformer **1-7** at the B3LYP/6-31G(d) level of theory in MeOH with IEFPCM solvent model.

| ***Num^a^*** | ***Transition^b^*** | ***CI-coeff^b^*** | ***ΔE (eV)^d^*** | ***λ (nm)^e^*** | ***f^f^*** | ***R_vel_^g^*** | ***R_len_^h^*** |
| --- | --- | --- | --- | --- | --- | --- | --- |
| 1 | 57->60 | 0.62682 | 4.0548 | 305.77 | 0.0001 | 0.2826 | -1.6505 |
| 2 | 59->60 | 0.68241 | 4.3471 | 285.21 | 0.0776 | -9.0339 | -6.4101 |
| 3 | 58->60 | 0.59083 | 5.3327 | 232.50 | 0.0922 | -8.2559 | -7.9582 |
|  | 59->61 | -0.30462 |  |  |  |  |  |
| 4 | 58->60 | 0.31114 | 6.0730 | 204.16 | 0.7826 | 30.7293 | 32.298 |
|  | 59->61 | 0.60106 |  |  |  |  |  |
| 5 | 56->60 | 0.58281 | 6.2879 | 197.18 | 0.0151 | -10.504 | -8.3184 |
| 6 | 55->60 | 0.53777 | 6.4675 | 191.70 | 0.0248 | 3.9681 | 4.9638 |
|  | 58->61 | 0.3279 |  |  |  |  |  |
| 7 | 55->60 | 0.3759 | 6.6385 | 186.76 | 0.0775 | -6.3143 | -7.483 |
|  | 56->60 | 0.30239 |  |  |  |  |  |
|  | 58->61 | -0.28848 |  |  |  |  |  |
|  | 59->62 | 0.22388 |  |  |  |  |  |
| 8 | 53->60 | 0.4571 | 6.8095 | 182.08 | 0.0852 | 19.2073 | 19.7468 |
|  | 54->60 | 0.34513 |  |  |  |  |  |
|  | 58->61 | -0.23391 |  |  |  |  |  |
| 9 | 57->61 | 0.64768 | 6.9442 | 178.54 | 0.0011 | 4.6374 | 3.5788 |
| 10 | 55->61 | -0.32518 | 7.2202 | 171.72 | 0.1310 | 20.9936 | 22.4338 |
|  | 56->61 | 0.2857 |  |  |  |  |  |
|  | 58->61 | 0.29808 |  |  |  |  |  |
|  | 59->62 | 0.41838 |  |  |  |  |  |
| 11 | 52->60 | 0.34269 | 7.3296 | 169.16 | 0.0383 | 12.2253 | 14.141 |
|  | 54->60 | 0.23431 |  |  |  |  |  |
|  | 57->62 | -0.28532 |  |  |  |  |  |
| 12 | 55->61 | 0.35079 | 7.4010 | 167.52 | 0.1284 | -34.5676 | -39.7399 |
|  | 56->61 | -0.24495 |  |  |  |  |  |
|  | 59->62 | 0.39734 |  |  |  |  |  |
| 13 | 51->60 | -0.23573 | 7.5509 | 164.20 | 0.0414 | -32.9026 | -33.8749 |
|  | 52->60 | -0.25234 |  |  |  |  |  |
|  | 53->60 | -0.29754 |  |  |  |  |  |
|  | 54->60 | 0.42755 |  |  |  |  |  |
| 14 | 52->60 | 0.42265 | 7.7163 | 160.68 | 0.0108 | 30.9975 | 30.2274 |
|  | 57->62 | 0.44687 |  |  |  |  |  |
| 15 | 49->60 | -0.28799 | 7.8547 | 157.85 | 0.0427 | -31.9071 | -31.8421 |
|  | 51->60 | 0.47644 |  |  |  |  |  |
| 16 | 56->61 | 0.30414 | 7.9090 | 156.76 | 0.0076 | 15.4889 | 10.1468 |
|  | 59->63 | -0.30233 |  |  |  |  |  |
| 17 | 49->60 | 0.33947 | 7.9590 | 155.78 | 0.0109 | 15.2547 | 18.1484 |
|  | 51->60 | 0.36443 |  |  |  |  |  |
|  | 53->61 | 0.28159 |  |  |  |  |  |
| 18 | 56->61 | 0.27532 | 8.0227 | 154.54 | 0.0372 | 15.1692 | 15.1748 |
|  | 58->62 | 0.32958 |  |  |  |  |  |
|  | 59->63 | 0.3557 |  |  |  |  |  |
|  | 59->64 | -0.23345 |  |  |  |  |  |
| 19 | 58->62 | 0.36443 | 8.1126 | 152.83 | 0.0352 | -13.7992 | -18.502 |
|  | 59->64 | 0.33357 |  |  |  |  |  |
| 20 | 55->61 | 0.29883 | 8.1463 | 152.20 | 0.0384 | 38.9854 | 36.1629 |
|  | 56->61 | 0.25227 |  |  |  |  |  |
|  | 58->62 | -0.2862 |  |  |  |  |  |
|  | 59->64 | 0.30327 |  |  |  |  |  |
| 21 | 49->60 | -0.30927 | 8.2354 | 150.55 | 0.0304 | 15.2239 | 16.6933 |
|  | 53->61 | 0.34981 |  |  |  |  |  |
|  | 54->61 | 0.28492 |  |  |  |  |  |
| 22 | 55->64 | 0.23591 | 8.3269 | 148.90 | 0.0144 | -28.5168 | -27.4306 |
|  | 59->65 | -0.35783 |  |  |  |  |  |
|  | 59->66 | 0.37209 |  |  |  |  |  |
| 23 | 56->63 | -0.23901 | 8.4788 | 146.23 | 0.0079 | 5.4723 | -1.197 |
|  | 56->65 | 0.27396 |  |  |  |  |  |
|  | 59->63 | -0.22864 |  |  |  |  |  |
| 24 | 47->60 | 0.25475 | 8.5313 | 145.33 | 0.0383 | -29.028 | -31.6805 |
|  | 50->60 | 0.46869 |  |  |  |  |  |
| 25 | 52->61 | 0.2842 | 8.5494 | 145.02 | 0.0005 | 9.0418 | -0.892 |
|  | 59->63 | 0.27705 |  |  |  |  |  |
| 26 | 52->61 | 0.38009 | 8.6044 | 144.09 | 0.0195 | -19.9949 | -12.4409 |
|  | 59->64 | -0.27461 |  |  |  |  |  |
|  | 59->65 | -0.29925 |  |  |  |  |  |
| 27 | 47->60 | 0.23649 | 8.6151 | 143.92 | 0.0648 | 15.5992 | 18.5824 |
|  | 48->60 | 0.40395 |  |  |  |  |  |
|  | 50->60 | -0.27175 |  |  |  |  |  |
| 28 | 59->66 | 0.44007 | 8.8125 | 140.69 | 0.0075 | -4.7985 | -4.7674 |
| 29 | 51->61 | 0.32661 | 8.8713 | 139.76 | 0.0296 | 13.6921 | 13.0198 |
|  | 54->61 | -0.23835 |  |  |  |  |  |
|  | 59->67 | -0.30482 |  |  |  |  |  |
| 30 | 57->63 | 0.29009 | 8.9166 | 139.05 | 0.0008 | -3.779 | -6.1608 |
|  | 59->68 | -0.28837 |  |  |  |  |  |

*^a^*Number of the excited states; *^b^*Only transitions with contribution over 10.0% were listed; *^c^*Configuration-interaction coefficient; *^d^*Excitation energy; *^e^*Wavelength; *^f^*Oscillator strength; *^g^*Rotatory strength in velocity form (10^-40^ cgs); *^h^*Rotatory strength in length form (10^-40^ cgs).

**Table S10**. Key transitions, oscillator strengths, and rotatory strengths in the ECD spectrum of conformer **1-8** at the B3LYP/6-31G(d) level of theory in MeOH with IEFPCM solvent model.

| ***Num^a^*** | ***Transition^b^*** | ***CI-coeff^b^*** | ***ΔE (eV)^d^*** | ***λ (nm)^e^*** | ***f^f^*** | ***R_vel_^g^*** | ***R_len_^h^*** |
| --- | --- | --- | --- | --- | --- | --- | --- |
| 1 | 58->60 | 0.62499 | 4.0563 | 305.66 | 0.0004 | 8.1496 | 10.8422 |
| 2 | 59->60 | 0.68202 | 4.3394 | 285.72 | 0.0829 | 4.4372 | 2.0648 |
| 3 | 57->60 | 0.60432 | 5.3613 | 231.26 | 0.0965 | 6.9115 | 6.361 |
|  | 59->61 | 0.30525 |  |  |  |  |  |
| 4 | 57->60 | -0.31384 | 6.0863 | 203.71 | 0.7741 | -54.0292 | -54.8152 |
|  | 59->61 | 0.60945 |  |  |  |  |  |
| 5 | 55->60 | 0.5229 | 6.4391 | 192.55 | 0.0012 | 6.8323 | 6.1388 |
|  | 56->60 | -0.42436 |  |  |  |  |  |
| 6 | 54->60 | 0.31503 | 6.4960 | 190.86 | 0.0692 | 1.1171 | 0.8975 |
|  | 57->61 | 0.48454 |  |  |  |  |  |
|  | 59->62 | 0.30343 |  |  |  |  |  |
| 7 | 53->60 | 0.32656 | 6.7289 | 184.26 | 0.0162 | -12.2563 | -12.8416 |
|  | 55->60 | 0.29836 |  |  |  |  |  |
|  | 56->60 | 0.42105 |  |  |  |  |  |
| 8 | 53->60 | 0.23148 | 6.8487 | 181.03 | 0.0583 | -2.8877 | -3.1478 |
|  | 54->60 | 0.36237 |  |  |  |  |  |
|  | 56->60 | -0.28915 |  |  |  |  |  |
| 9 | 58->61 | 0.64437 | 6.9717 | 177.84 | 0.0072 | 10.3422 | 11.126 |
| 10 | 53->60 | 0.40897 | 7.2935 | 169.99 | 0.0325 | -4.2128 | -4.9097 |
|  | 54->60 | -0.37643 |  |  |  |  |  |
|  | 59->62 | 0.24984 |  |  |  |  |  |
| 11 | 57->61 | -0.37182 | 7.3182 | 169.42 | 0.2691 | -0.1265 | 2.0924 |
|  | 59->62 | 0.46035 |  |  |  |  |  |
| 12 | 55->61 | 0.43717 | 7.4207 | 167.08 | 0.0549 | 25.116 | 23.0951 |
|  | 56->61 | -0.31616 |  |  |  |  |  |
|  | 59->62 | 0.26647 |  |  |  |  |  |
| 13 | 58->62 | 0.44865 | 7.5621 | 163.95 | 0.0086 | -27.5206 | -27.1809 |
| 14 | 50->60 | -0.30255 | 7.8446 | 158.05 | 0.0335 | 45.6616 | 48.6506 |
|  | 51->60 | 0.31608 |  |  |  |  |  |
|  | 53->60 | 0.23498 |  |  |  |  |  |
|  | 58->62 | 0.26023 |  |  |  |  |  |
| 15 | 51->60 | 0.4521 | 7.8735 | 157.47 | 0.0423 | 1.6777 | 1.531 |
|  | 52->60 | 0.34554 |  |  |  |  |  |
| 16 | 59->64 | 0.42041 | 8.0119 | 154.75 | 0.0439 | -62.2297 | -56.0485 |
| 17 | 50->60 | -0.26913 | 8.0737 | 153.57 | 0.0387 | -10.0982 | -11.5951 |
|  | 54->61 | 0.28992 |  |  |  |  |  |
|  | 59->64 | 0.27941 |  |  |  |  |  |
| 18 | 50->60 | 0.30262 | 8.1021 | 153.03 | 0.0190 | 14.6798 | 14.6079 |
|  | 57->62 | 0.45594 |  |  |  |  |  |
| 19 | 59->63 | 0.49942 | 8.1484 | 152.16 | 0.0066 | -5.4693 | -8.9434 |
| 20 | 50->60 | 0.31533 | 8.1836 | 151.50 | 0.1039 | 35.8454 | 37.6598 |
|  | 52->60 | -0.26594 |  |  |  |  |  |
|  | 53->61 | 0.25042 |  |  |  |  |  |
|  | 57->62 | -0.25511 |  |  |  |  |  |
| 21 | 59->65 | 0.44287 | 8.2450 | 150.37 | 0.0015 | 12.2604 | 3.4459 |
|  | 59->66 | -0.31843 |  |  |  |  |  |
| 22 | 48->60 | 0.40162 | 8.3343 | 148.76 | 0.0030 | 6.169 | 6.7493 |
|  | 50->60 | 0.25088 |  |  |  |  |  |
| 23 | 56->63 | 0.25281 | 8.3489 | 148.50 | 0.0061 | -19.9746 | -27.0156 |
| 24 | 54->61 | -0.29887 | 8.3758 | 148.03 | 0.0252 | 43.1002 | 45.6635 |
|  | 56->61 | 0.33753 |  |  |  |  |  |
| 25 | 47->60 | 0.34495 | 8.4956 | 145.94 | 0.0127 | -9.313 | -8.9182 |
|  | 53->61 | -0.25087 |  |  |  |  |  |
| 26 | 54->61 | 0.32054 | 8.5293 | 145.36 | 0.0150 | -3.3502 | -3.4069 |
|  | 55->61 | 0.24805 |  |  |  |  |  |
|  | 56->61 | 0.22909 |  |  |  |  |  |
| 27 | 55->64 | 0.23903 | 8.6940 | 142.61 | 0.0313 | -15.4976 | -18.3128 |
|  | 59->64 | 0.32541 |  |  |  |  |  |
| 28 | 59->65 | 0.35763 | 8.7309 | 142.01 | 0.0053 | -1.6023 | -0.9358 |
|  | 59->66 | 0.36341 |  |  |  |  |  |
|  | 59->67 | -0.30901 |  |  |  |  |  |
| 29 | 58->63 | 0.54388 | 8.8882 | 139.49 | 0.0225 | 22.1827 | 17.6867 |
|  | 58->65 | 0.24344 |  |  |  |  |  |
| 30 | 57->64 | 0.33783 | 8.9712 | 138.20 | 0.0046 | -1.5207 | 1.2668 |
|  | 57->65 | -0.23584 |  |  |  |  |  |
|  | 57->66 | 0.2277 |  |  |  |  |  |
|  | 59->67 | -0.28332 |  |  |  |  |  |

*^a^*Number of the excited states; *^b^*Only transitions with contribution over 10.0% were listed; *^c^*Configuration-interaction coefficient; *^d^*Excitation energy; *^e^*Wavelength; *^f^*Oscillator strength; *^g^*Rotatory strength in velocity form (10^-40^ cgs); *^h^*Rotatory strength in length form (10^-40^ cgs).

**Table S11.** Experimental and calculated ^13^C-NMR chemical shifts of **2**

| No. | δexptl. | *SRR*--δcalcd. | *SRS*-δcalcd. | *SSR*-δcalcd. | *SSS*-δcalcd. |
| --- | --- | --- | --- | --- | --- |
| 1 | 205.2 | 209.7 | 211.0 | 212.4 | 212.8 |
| 2 | 46.6 | 46.2 | 45.3 | 44.6 | 45.0 |
| 3 | 69.9 | 72.4 | 73.8 | 74.8 | 75.6 |
| 4 | 28.7 | 27.5 | 27.0 | 26.9 | 26.7 |
| 5 | 34.1 | 36.5 | 36.8 | 38.2 | 38.2 |
| 6 | 83.3 | 79.5 | 79.2 | 80.5 | 79.9 |
| 7 | 20.8 | 21.9 | 20.6 | 20.7 | 20.8 |
| 8 | 130.7 | 129.8 | 130.2 | 130.5 | 130.6 |
| 9 | 31.2 | 34.1 | 31.0 | 30.8 | 33.2 |
| 10 | 65.8 | 69.3 | 68.5 | 69.0 | 70.1 |
| 11 | 29.6 | 31.0 | 31.3 | 31.6 | 29.7 |
| 12 | 31.8 | 35.6 | 33.3 | 34.8 | 36.2 |
| 13 | 155.4 | 157.7 | 160.2 | 159.7 | 159.7 |
| 14 | 18.0 | 19.2 | 18.9 | 18.8 | 19.1 |
| 15 | 190.8 | 186.1 | 185.3 | 185.3 | 185.0 |
| 16 | 170.3 | 168.3 | 168.4 | 168.5 | 168.4 |
| 17 | 21.2 | 22.1 | 22.1 | 21.9 | 22.0 |


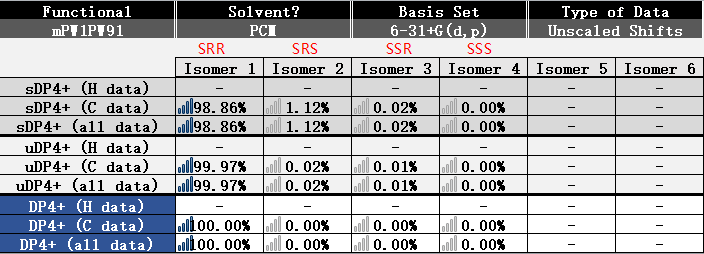


**Table S12.** Conformational analysis of the B3LYP/6-31G(d) optimized conformers of **2**-*SRR* in the gas phase (T=298.15 K)

| **Conformer** | **E*^a^* (Hartree)** | **C*^b^* (Hartree)** | **G*^c^* (kcal/mol)** | **ΔG*^d^* (kcal/mol)** | **Population*^e^*** |
| --- | --- | --- | --- | --- | --- |
| **2-1** | -1038.278668 | 0.330686 | -651312.358881 | 0.0 | 75.69% |
| **2-2** | -1038.275345 | 0.329238 | -651311.182149 | 1.176732 | 10.37% |
| **2-3** | -1038.280938 | 0.335089 | -651311.020404 | 1.338476 | 7.89% |
| **2-4** | -1038.274941 | 0.329344 | -651310.862299 | 1.496581 | 6.04% |

*^a^*Electronic energy obtained at M062X/6-311+G(2d,p) level of theory; *^b^*Thermal correction to Gibbs free energy obtained at B3LYP/6-31G(d) level of theory; *^c^*Gibbs free energy (E + C); *^d^*The relative Gibbs free energy; *^e^*The Boltzmann distribution of each conformer.

**Table S13.** Atomic coordinates (Å) of **2-1** obtained at the B3LYP/6-31G(d) level of theory in the gas phase.

| C | -0.967243 | -0.782614 | 1.440696 | H | -2.380222 | 0.663606 | 2.142498 |
| --- | --- | --- | --- | --- | --- | --- | --- |
| C | -1.985857 | 0.318969 | 1.183635 | H | -3.740036 | -0.885471 | 0.752463 |
| C | -3.101911 | -0.151905 | 0.250011 | H | -2.001057 | 0.021719 | -1.586629 |
| C | -2.544641 | -0.752474 | -1.035990 | H | -3.372819 | -1.101727 | -1.659042 |
| C | -1.600455 | -1.914588 | -0.711920 | H | -1.187515 | -2.336411 | -1.634386 |
| C | -0.421259 | -1.497842 | 0.183510 | H | -2.152972 | -2.717591 | -0.206579 |
| C | 0.439918 | -2.699369 | 0.556242 | H | 1.253721 | -2.420795 | 1.228821 |
| C | 3.985633 | 0.210130 | -0.366723 | H | -0.165609 | -3.447916 | 1.075837 |
| C | 2.715248 | -0.558375 | -0.665253 | H | 0.857269 | -3.148340 | -0.349676 |
| C | 1.476561 | 0.055216 | -0.018158 | H | 2.852856 | -1.596459 | -0.352412 |
| C | 1.457746 | 1.558039 | -0.273720 | H | 2.549176 | -0.598490 | -1.750355 |
| C | 2.688176 | 2.217321 | 0.353817 | H | 1.494861 | -0.120589 | 1.065715 |
| C | 3.985472 | 1.479414 | 0.106880 | H | 0.543113 | 2.003350 | 0.130894 |
| C | 5.206110 | 2.290754 | 0.456736 | H | 1.446255 | 1.724758 | -1.357986 |
| C | 5.226944 | -0.534531 | -0.645220 | H | 2.553513 | 2.305332 | 1.443704 |
| C | -5.192788 | 0.790922 | -0.404553 | H | 2.790853 | 3.250256 | -0.005887 |
| C | -5.901627 | 2.094909 | -0.679329 | H | 5.283640 | 3.158935 | -0.211257 |
| O | 0.289429 | -0.512933 | -0.588720 | H | 5.102826 | 2.691670 | 1.473546 |
| O | -5.686135 | -0.311347 | -0.498861 | H | 6.146431 | 1.742370 | 0.408652 |
| O | -0.581104 | -1.053301 | 2.561352 | H | 6.183511 | -0.005880 | -0.471785 |
| O | 5.238763 | -1.682023 | -1.061346 | H | -5.852464 | 2.744642 | 0.200119 |
| O | -3.908674 | 1.016677 | -0.034237 | H | -5.406482 | 2.620898 | -1.502307 |
| H | -1.472078 | 1.156488 | 0.696716 | H | -6.941172 | 1.895204 | -0.940130 |

**Table S14.** Atomic coordinates (Å) of **2-2** obtained at the B3LYP/6-31G(d) level of theory in the gas phase.

| C | 1.160640 | 1.949457 | 0.649753 | H | 2.791402 | 1.224428 | 1.830845 |
| --- | --- | --- | --- | --- | --- | --- | --- |
| C | 2.098148 | 0.831862 | 1.083286 | H | 3.562846 | 0.937602 | -0.515588 |
| C | 2.837981 | 0.225014 | -0.109673 | H | 1.232155 | -1.007959 | -0.826463 |
| C | 1.875273 | -0.208932 | -1.209340 | H | 2.447680 | -0.604996 | -2.052859 |
| C | 1.017210 | 0.975968 | -1.664155 | H | 0.309510 | 0.656177 | -2.436414 |
| C | 0.207750 | 1.608309 | -0.519164 | H | 1.654668 | 1.751441 | -2.108862 |
| C | -0.548196 | 2.843172 | -0.997325 | H | -1.231208 | 2.567126 | -1.806325 |
| C | -3.595990 | -0.298013 | -0.020541 | H | -1.114336 | 3.309056 | -0.187926 |
| C | -2.992472 | 1.007551 | 0.465233 | H | 0.155373 | 3.592831 | -1.370698 |
| C | -1.571737 | 0.847091 | 1.007279 | H | -3.619770 | 1.445353 | 1.256374 |
| C | -1.534417 | -0.337855 | 1.969780 | H | -2.991172 | 1.735924 | -0.352788 |
| C | -1.898011 | -1.628885 | 1.234528 | H | -1.263162 | 1.762892 | 1.523280 |
| C | -3.092242 | -1.510575 | 0.318760 | H | -2.253885 | -0.150522 | 2.776609 |
| C | -3.634622 | -2.820350 | -0.188826 | H | -0.545855 | -0.427910 | 2.431741 |
| C | -4.760185 | -0.147780 | -0.911319 | H | -2.082143 | -2.437925 | 1.953932 |
| C | 4.667431 | -1.293969 | -0.294270 | H | -1.046079 | -1.963198 | 0.624661 |
| C | 5.327666 | -2.481489 | 0.362708 | H | -2.922483 | -3.628899 | 0.007979 |
| O | -0.660161 | 0.555303 | -0.066225 | H | -4.579134 | -3.069049 | 0.310532 |
| O | 5.047158 | -0.747456 | -1.306244 | H | -3.864751 | -2.784540 | -1.255420 |
| O | 1.137681 | 3.027644 | 1.210922 | H | -4.978711 | 0.910938 | -1.183793 |
| O | -5.471835 | -1.038363 | -1.348936 | H | 5.607787 | -2.234758 | 1.391754 |
| O | 3.573260 | -0.909460 | 0.408268 | H | 4.626132 | -3.320918 | 0.407598 |
| H | 1.498764 | 0.038893 | 1.546401 | H | 6.212188 | -2.766802 | -0.207146 |

**Table S15.** Atomic coordinates (Å) of **2-3** obtained at the B3LYP/6-31G(d) level of theory in the gas phase.

| C | -0.382477 | 0.874579 | -1.187912 | H | -2.125662 | 1.435166 | -2.266845 |
| --- | --- | --- | --- | --- | --- | --- | --- |
| C | -1.852480 | 0.665293 | -1.530116 | H | -3.834477 | 0.740700 | -0.683425 |
| C | -2.799424 | 0.790180 | -0.337831 | H | -3.228597 | 2.115461 | 1.303681 |
| C | -2.530285 | 2.059672 | 0.461730 | H | -2.733745 | 2.924685 | -0.181145 |
| C | -1.089459 | 2.080316 | 0.983765 | H | -0.986732 | 1.249610 | 1.685080 |
| C | -0.032820 | 1.957254 | -0.132451 | H | -0.892669 | 3.000959 | 1.545383 |
| C | 0.075747 | 3.272777 | -0.926301 | H | 0.345341 | 4.082180 | -0.241107 |
| C | 1.865781 | -1.366349 | 0.147247 | H | 0.859989 | 3.172753 | -1.681269 |
| C | 1.043610 | -0.605486 | 1.162597 | H | -0.863016 | 3.531530 | -1.425368 |
| C | 1.531738 | 0.821348 | 1.424542 | H | 1.056920 | -1.142054 | 2.122290 |
| C | 3.050316 | 0.814839 | 1.574287 | H | -0.004922 | -0.624989 | 0.854090 |
| C | 3.702319 | 0.310576 | 0.285631 | H | 1.071018 | 1.187268 | 2.352357 |
| C | 3.089870 | -0.955333 | -0.261833 | H | 3.314943 | 0.162826 | 2.415964 |
| C | 3.936246 | -1.619456 | -1.314360 | H | 3.406550 | 1.823109 | 1.808741 |
| C | 1.214577 | -2.561505 | -0.395570 | H | 4.780231 | 0.162060 | 0.433069 |
| C | -3.183533 | -1.495358 | 0.260088 | H | 3.600469 | 1.078838 | -0.493449 |
| C | -2.839839 | -2.553415 | 1.271431 | H | 3.487422 | -2.497385 | -1.777705 |
| O | 1.281259 | 1.761911 | 0.373001 | H | 4.151998 | -0.895194 | -2.110853 |
| O | -3.880960 | -1.638558 | -0.719263 | H | 4.904619 | -1.914110 | -0.888964 |
| O | 0.491952 | 0.302079 | -1.805591 | H | 1.771102 | -3.141887 | -1.152830 |
| O | 0.103091 | -2.944753 | -0.053310 | H | -2.919693 | -2.156309 | 2.287449 |
| O | -2.581526 | -0.313443 | 0.575962 | H | -3.504607 | -3.408115 | 1.140840 |
| H | -1.963431 | -0.301973 | -2.024766 | H | -1.802905 | -2.862616 | 1.102719 |

**Table S16.** Atomic coordinates (Å) of **2-4** obtained at the B3LYP/6-31G(d) level of theory in the gas phase.

| C | -1.283047 | 1.722444 | -0.589315 | H | -3.010298 | 0.780785 | -1.431505 |
| --- | --- | --- | --- | --- | --- | --- | --- |
| C | -2.210312 | 0.525223 | -0.734617 | H | -3.491556 | 0.824323 | 0.976791 |
| C | -2.774483 | 0.078533 | 0.619842 | H | -1.034602 | -0.972293 | 1.311489 |
| C | -1.672256 | -0.146949 | 1.643524 | H | -2.116717 | -0.436159 | 2.601849 |
| C | -0.829492 | 1.121927 | 1.809951 | H | -0.024544 | 0.948504 | 2.532111 |
| C | -0.187120 | 1.593109 | 0.494068 | H | -1.450045 | 1.937644 | 2.203622 |
| C | 0.557919 | 2.909399 | 0.688100 | H | 1.000777 | 3.263783 | -0.245061 |
| C | 3.630977 | -0.210870 | -0.159402 | H | -0.133861 | 3.684634 | 1.030173 |
| C | 2.895789 | 0.962573 | -0.781416 | H | 1.344173 | 2.782237 | 1.438413 |
| C | 1.434415 | 0.650897 | -1.106453 | H | 3.398492 | 1.278449 | -1.708038 |
| C | 1.361025 | -0.684038 | -1.844617 | H | 2.944846 | 1.823429 | -0.106160 |
| C | 1.887644 | -1.810869 | -0.953698 | H | 1.010643 | 1.449971 | -1.724804 |
| C | 3.167575 | -1.483679 | -0.223600 | H | 1.969104 | -0.609654 | -2.754790 |
| C | 3.843247 | -2.661530 | 0.426929 | H | 0.333316 | -0.895137 | -2.158027 |
| C | 4.876975 | 0.142658 | 0.543688 | H | 2.040936 | -2.723858 | -1.544448 |
| C | -4.756557 | -1.106489 | 0.016896 | H | 1.132779 | -2.071019 | -0.197446 |
| C | -5.356021 | -2.487445 | -0.089958 | H | 4.720827 | -2.973337 | -0.153229 |
| O | 0.673057 | 0.508830 | 0.105313 | H | 4.216372 | -2.423933 | 1.424654 |
| O | -5.320837 | -0.070317 | -0.255750 | H | 3.154670 | -3.511651 | 0.481868 |
| O | -1.373815 | 2.703165 | -1.301636 | H | 5.054393 | 1.241345 | 0.608995 |
| O | 5.691621 | -0.625748 | 1.030423 | H | -4.787439 | -3.085254 | -0.810006 |
| O | -3.478320 | -1.176379 | 0.464807 | H | -5.299432 | -2.999392 | 0.875773 |
| H | -1.629744 | -0.310320 | -1.144302 | H | -6.394373 | -2.408856 | -0.412703 |

**Table S17.** Key transitions, oscillator strengths, and rotatory strengths in the ECD spectrum of conformer **2-1** at the Cam-B3LYP/Def2SVP level of theory in MeOH with IEFPCM solvent model.

| ***Num^a^*** | ***Transition^b^*** | ***CI-coeff^b^*** | ***ΔE (eV)^d^*** | ***λ (nm)^e^*** | ***f^f^*** | ***R_vel_^g^*** | ***R_len_^h^*** |
| --- | --- | --- | --- | --- | --- | --- | --- |
| 1 | 81->84 | 0.6522 | 3.9616 | 312.97 | 0.0004 | 5.2695 | 4.4642 |
| 2 | 82->85 | -0.31551 | 4.1493 | 298.81 | 0.0007 | 3.4325 | 0.9713 |
|  | 83->85 | 0.5354 |  |  |  |  |  |
| 3 | 82->84 | 0.60493 | 5.4693 | 226.69 | 0.4115 | 16.734 | 11.8156 |
|  | 83->84 | 0.34459 |  |  |  |  |  |
| 4 | 79->86 | 0.55729 | 5.9516 | 208.32 | 0.0016 | -4.535 | -4.8438 |
|  | 80->86 | -0.36498 |  |  |  |  |  |
| 5 | 80->84 | -0.28764 | 6.1920 | 200.23 | 0.0001 | 0.6205 | 0.9078 |
|  | 82->84 | -0.29409 |  |  |  |  |  |
|  | 83->84 | 0.50827 |  |  |  |  |  |
| 6 | 79->85 | 0.27069 | 6.2950 | 196.96 | 0.0064 | 6.8283 | 6.8185 |
|  | 80->85 | 0.52568 |  |  |  |  |  |
|  | 83->85 | -0.28926 |  |  |  |  |  |
| 7 | 74->84 | -0.24234 | 6.9812 | 177.60 | 0.0004 | -4.7817 | -4.1909 |
|  | 80->84 | 0.32021 |  |  |  |  |  |
|  | 83->84 | 0.24066 |  |  |  |  |  |
| 8 | 82->85 | 0.52905 | 7.1237 | 174.04 | 0.0129 | 1.9508 | 2.083 |
|  | 83->85 | 0.25968 |  |  |  |  |  |
| 9 | 73->84 | -0.23398 | 7.1255 | 174.00 | 0.0024 | 15.9386 | 16.5784 |
|  | 76->84 | 0.39505 |  |  |  |  |  |
|  | 80->84 | -0.29714 |  |  |  |  |  |
| 10 | 77->85 | 0.36246 | 7.2149 | 171.84 | 0.0255 | 1.2649 | 1.3389 |
|  | 78->85 | -0.31807 |  |  |  |  |  |
|  | 79->85 | -0.30086 |  |  |  |  |  |
|  | 82->85 | -0.28679 |  |  |  |  |  |
| 11 | 74->84 | 0.34756 | 7.5533 | 164.15 | 0.0008 | -4.3905 | -4.3056 |
|  | 80->84 | 0.25286 |  |  |  |  |  |
| 12 | 77->85 | 0.43962 | 7.6431 | 162.22 | 0.0023 | -3.7377 | -3.3926 |
|  | 78->85 | 0.37316 |  |  |  |  |  |
|  | 81->85 | 0.30973 |  |  |  |  |  |
| 13 | 78->85 | -0.2397 | 7.6924 | 161.18 | 0.0067 | 0.0915 | 0.0184 |
|  | 81->85 | 0.57763 |  |  |  |  |  |
| 14 | 76->84 | 0.26736 | 7.8200 | 158.55 | 0.0258 | -9.5651 | -10.389 |
|  | 77->84 | 0.48632 |  |  |  |  |  |
| 15 | 81->89 | 0.44942 | 7.9660 | 155.64 | 0.0051 | -22.94 | -24.19 |
| 16 | 76->85 | 0.23093 | 7.9920 | 155.14 | 0.0079 | -0.4503 | -0.666 |
|  | 79->85 | 0.38847 |  |  |  |  |  |
|  | 80->85 | -0.31851 |  |  |  |  |  |
| 17 | 75->85 | 0.33438 | 8.1320 | 152.46 | 0.0883 | -22.0266 | -18.2629 |
|  | 76->85 | 0.32304 |  |  |  |  |  |
|  | 78->86 | -0.33463 |  |  |  |  |  |
|  | 79->85 | -0.25197 |  |  |  |  |  |
| 18 | 78->86 | 0.46376 | 8.1611 | 151.92 | 0.1047 | 10.1683 | 11.1907 |
| 19 | 82->87 | 0.41904 | 8.1710 | 151.74 | 0.0735 | 79.7924 | 72.6466 |
|  | 82->89 | -0.30514 |  |  |  |  |  |
|  | 83->87 | 0.32675 |  |  |  |  |  |
| 20 | 82->86 | -0.27247 | 8.2157 | 150.91 | 0.0123 | 14.0524 | 16.2521 |
|  | 83->86 | 0.50735 |  |  |  |  |  |
| 21 | 79->84 | 0.54278 | 8.2742 | 149.85 | 0.0053 | 1.9644 | 2.5552 |
|  | 80->84 | -0.26772 |  |  |  |  |  |
| 22 | 82->87 | -0.27336 | 8.2848 | 149.65 | 0.0036 | -23.9907 | -21.2729 |
|  | 83->87 | 0.36212 |  |  |  |  |  |
| 23 | 74->85 | -0.3135 | 8.3436 | 148.60 | 0.0227 | 17.5257 | 16.5646 |
|  | 75->85 | 0.42792 |  |  |  |  |  |
|  | 76->85 | -0.27656 |  |  |  |  |  |
| 24 | 73->85 | -0.26375 | 8.4426 | 146.86 | 0.0040 | 3.8264 | 2.8857 |
|  | 74->85 | 0.31592 |  |  |  |  |  |
|  | 78->85 | 0.29065 |  |  |  |  |  |
| 25 | 82->87 | 0.31226 | 8.4486 | 146.75 | 0.0747 | -46.3199 | -40.6307 |
|  | 82->89 | 0.46098 |  |  |  |  |  |
|  | 83->89 | 0.24069 |  |  |  |  |  |
| 26 | 69->84 | 0.26256 | 8.5408 | 145.17 | 0.0363 | 17.9158 | 19.399 |
|  | 70->84 | 0.23863 |  |  |  |  |  |
|  | 71->84 | 0.32618 |  |  |  |  |  |
| 27 | 78->84 | 0.58026 | 8.6064 | 144.06 | 0.0066 | -5.4409 | -5.4694 |
| 28 | 82->88 | -0.26499 | 8.6422 | 143.46 | 0.0201 | -3.4882 | -5.0612 |
|  | 83->88 | 0.41559 |  |  |  |  |  |
|  | 83->91 | 0.24433 |  |  |  |  |  |
| 29 | 68->84 | 0.29659 | 8.6544 | 143.26 | 0.0128 | -20.0957 | -21.3172 |
|  | 76->84 | -0.2308 |  |  |  |  |  |
|  | 77->84 | 0.28809 |  |  |  |  |  |
|  | 78->84 | 0.35437 |  |  |  |  |  |
| 30 | 69->86 | 0.23965 | 8.7022 | 142.47 | 0.0021 | -3.495 | -4.1382 |
|  | 72->86 | 0.23087 |  |  |  |  |  |
|  | 74->86 | -0.27087 |  |  |  |  |  |
|  | 75->86 | 0.35518 |  |  |  |  |  |

*^a^*Number of the excited states; *^b^*Only transitions with contribution over 10.0% were listed; *^c^*Configuration-interaction coefficient; *^d^*Excitation energy; *^e^*Wavelength; *^f^*Oscillator strength; *^g^*Rotatory strength in velocity form (10^-40^ cgs); *^h^*Rotatory strength in length form (10^-40^ cgs).

**Table S18**. Key transitions, oscillator strengths, and rotatory strengths in the ECD spectrum of conformer **2-2** at the Cam-B3LYP/Def2SVP level of theory in MeOH with IEFPCM solvent model.

| ***Num^a^*** | ***Transition^b^*** | ***CI-coeff^b^*** | ***ΔE (eV)^d^*** | ***λ (nm)^e^*** | ***f^f^*** | ***R_vel_^g^*** | ***R_len_^h^*** |
| --- | --- | --- | --- | --- | --- | --- | --- |
| 1 | 81->84 | 0.6272 | 3.8065 | 325.71 | 0.0003 | -3.1266 | -2.5916 |
|  | 82->84 | -0.2461 |  |  |  |  |  |
| 2 | 81->85 | 0.25101 | 4.1443 | 299.17 | 0.0006 | -0.7506 | -3.3442 |
|  | 82->85 | 0.58303 |  |  |  |  |  |
| 3 | 83->84 | 0.68312 | 5.1987 | 238.49 | 0.2750 | -10.6437 | -12.6925 |
| 4 | 79->86 | 0.52092 | 5.9522 | 208.30 | 0.0017 | -4.3005 | -4.8447 |
|  | 80->86 | 0.41286 |  |  |  |  |  |
| 5 | 80->84 | -0.27781 | 6.0907 | 203.56 | 0.0059 | 9.5422 | 11.8977 |
|  | 82->84 | 0.5595 |  |  |  |  |  |
| 6 | 79->85 | -0.31322 | 6.2898 | 197.12 | 0.0040 | 4.7615 | 4.7002 |
|  | 80->85 | 0.49512 |  |  |  |  |  |
|  | 82->85 | -0.22713 |  |  |  |  |  |
|  | 83->85 | 0.22618 |  |  |  |  |  |
| 7 | 76->84 | -0.33711 | 6.7842 | 182.75 | 0.0008 | -5.2449 | -5.4428 |
|  | 77->84 | 0.46992 |  |  |  |  |  |
| 8 | 83->85 | 0.61856 | 6.9491 | 178.42 | 0.0075 | 2.7135 | 2.9389 |
| 9 | 74->84 | 0.28654 | 7.0206 | 176.60 | 0.0003 | 4.4559 | 4.6124 |
|  | 79->84 | -0.26453 |  |  |  |  |  |
|  | 80->84 | 0.34379 |  |  |  |  |  |
|  | 82->84 | 0.24317 |  |  |  |  |  |
| 10 | 76->85 | 0.23028 | 7.1890 | 172.46 | 0.0282 | -3.4596 | -3.9303 |
|  | 77->85 | 0.34828 |  |  |  |  |  |
|  | 78->85 | 0.32715 |  |  |  |  |  |
|  | 79->85 | -0.34387 |  |  |  |  |  |
| 11 | 81->85 | 0.41256 | 7.4881 | 165.58 | 0.0019 | -13.9079 | -14.2249 |
| 12 | 74->84 | 0.24878 | 7.5172 | 164.93 | 0.0052 | -6.4292 | -8.421 |
|  | 80->84 | -0.23757 |  |  |  |  |  |
|  | 81->85 | 0.3339 |  |  |  |  |  |
| 13 | 76->85 | -0.27127 | 7.6900 | 161.23 | 0.0044 | -2.2986 | -2.6442 |
|  | 77->85 | -0.32463 |  |  |  |  |  |
|  | 78->85 | 0.38065 |  |  |  |  |  |
|  | 81->85 | -0.33086 |  |  |  |  |  |
| 14 | 74->84 | 0.25628 | 7.8791 | 157.36 | 0.0205 | 9.8858 | 14.0078 |
|  | 81->88 | 0.30871 |  |  |  |  |  |
|  | 83->88 | 0.23275 |  |  |  |  |  |
| 15 | 76->84 | 0.2929 | 7.9295 | 156.36 | 0.0219 | -12.2183 | -5.8902 |
|  | 77->84 | 0.26153 |  |  |  |  |  |
|  | 79->84 | -0.25589 |  |  |  |  |  |
| 16 | 79->85 | 0.33604 | 7.9884 | 155.21 | 0.0338 | -20.9601 | -17.7232 |
|  | 80->85 | 0.33324 |  |  |  |  |  |
| 17 | 83->87 | 0.36176 | 8.0141 | 154.71 | 0.0426 | -37.9724 | -32.8195 |
| 18 | 82->87 | 0.47104 | 8.1041 | 152.99 | 0.0416 | 0.1115 | 0.2903 |
| 19 | 69->84 | 0.34959 | 8.1267 | 152.56 | 0.0685 | 8.7642 | 8.4765 |
|  | 78->86 | -0.30119 |  |  |  |  |  |
| 20 | 69->84 | 0.40037 | 8.1386 | 152.34 | 0.0652 | -82.5129 | -72.7297 |
|  | 78->86 | 0.31531 |  |  |  |  |  |
| 21 | 76->85 | -0.25969 | 8.1917 | 151.35 | 0.0664 | 43.317 | 51.7041 |
|  | 78->86 | 0.4256 |  |  |  |  |  |
| 22 | 82->86 | 0.52078 | 8.2278 | 150.69 | 0.0067 | -0.2057 | -0.2898 |
| 23 | 79->84 | 0.29052 | 8.2597 | 150.11 | 0.0060 | 10.22 | 8.8646 |
|  | 82->87 | 0.30435 |  |  |  |  |  |
| 24 | 79->84 | 0.28511 | 8.2851 | 149.65 | 0.0702 | 22.0316 | 15.0219 |
|  | 83->87 | -0.26053 |  |  |  |  |  |
|  | 83->88 | -0.25471 |  |  |  |  |  |
| 25 | 74->85 | 0.26923 | 8.3488 | 148.51 | 0.0264 | 10.8396 | 10.7091 |
|  | 75->85 | 0.43558 |  |  |  |  |  |
|  | 76->85 | -0.27512 |  |  |  |  |  |
| 26 | 71->84 | 0.33418 | 8.4062 | 147.49 | 0.0402 | 16.1656 | 16.7289 |
|  | 72->84 | 0.27442 |  |  |  |  |  |
|  | 78->84 | 0.24303 |  |  |  |  |  |
| 27 | 73->85 | 0.23895 | 8.4437 | 146.84 | 0.0038 | -7.3055 | -8.2323 |
|  | 74->85 | -0.25312 |  |  |  |  |  |
|  | 78->84 | 0.37519 |  |  |  |  |  |
| 28 | 78->84 | 0.50739 | 8.4895 | 146.04 | 0.0097 | 11.211 | 11.4327 |
|  | 78->85 | -0.22905 |  |  |  |  |  |
| 29 | 83->86 | 0.23044 | 8.5799 | 144.50 | 0.0062 | 33.1679 | 28.4941 |
|  | 83->88 | -0.31489 |  |  |  |  |  |
|  | 83->89 | 0.47125 |  |  |  |  |  |
| 30 | 82->88 | -0.27277 | 8.6735 | 142.95 | 0.0040 | 7.5747 | 7.4221 |
|  | 83->86 | 0.48973 |  |  |  |  |  |

*^a^*Number of the excited states; *^b^*Only transitions with contribution over 10.0% were listed; *^c^*Configuration-interaction coefficient; *^d^*Excitation energy; *^e^*Wavelength; *^f^*Oscillator strength; *^g^*Rotatory strength in velocity form (10^-40^ cgs); *^h^*Rotatory strength in length form (10^-40^ cgs).

**Table S19**. Key transitions, oscillator strengths, and rotatory strengths in the ECD spectrum of conformer **2-3** at the Cam-B3LYP/Def2SVP level of theory in MeOH with IEFPCM solvent model.

| ***Num^a^*** | ***Transition^b^*** | ***CI-coeff^b^*** | ***ΔE (eV)^d^*** | ***λ (nm)^e^*** | ***f^f^*** | ***R_vel_^g^*** | ***R_len_^h^*** |
| --- | --- | --- | --- | --- | --- | --- | --- |
| 1 | 81->84 | 0.63997 | 4.0134 | 308.92 | 0.0004 | -4.6552 | -4.1911 |
| 2 | 82->85 | 0.47325 | 4.2443 | 292.12 | 0.0012 | 18.9949 | 21.7197 |
|  | 83->85 | -0.3831 |  |  |  |  |  |
| 3 | 82->84 | 0.23653 | 5.3231 | 232.92 | 0.4114 | -18.3342 | -15.3723 |
|  | 83->84 | 0.6521 |  |  |  |  |  |
| 4 | 82->84 | 0.62113 | 5.7577 | 215.34 | 0.0144 | 9.848 | 11.2274 |
|  | 83->84 | -0.22496 |  |  |  |  |  |
| 5 | 79->86 | 0.67759 | 5.9707 | 207.65 | 0.0009 | 0.2949 | -0.0847 |
| 6 | 80->84 | -0.34304 | 6.1572 | 201.37 | 0.0041 | 9.0362 | 9.6671 |
|  | 80->85 | 0.37197 |  |  |  |  |  |
|  | 82->85 | 0.40541 |  |  |  |  |  |
| 7 | 80->84 | 0.45012 | 6.3884 | 194.08 | 0.0237 | -28.3507 | -31.1698 |
|  | 82->85 | 0.26099 |  |  |  |  |  |
|  | 83->85 | 0.3755 |  |  |  |  |  |
| 8 | 80->85 | 0.4339 | 6.5438 | 189.47 | 0.0159 | -15.9935 | -16.3587 |
|  | 83->85 | -0.39582 |  |  |  |  |  |
| 9 | 76->84 | 0.25624 | 6.8929 | 179.87 | 0.0006 | -8.4577 | -8.0589 |
|  | 78->84 | 0.51427 |  |  |  |  |  |
| 10 | 78->84 | -0.255 | 6.9620 | 178.09 | 0.0009 | 7.0977 | 7.5558 |
|  | 81->85 | 0.55221 |  |  |  |  |  |
| 11 | 75->84 | 0.50457 | 7.1223 | 174.08 | 0.0013 | 11.4761 | 11.4504 |
|  | 80->84 | -0.24204 |  |  |  |  |  |
|  | 81->85 | 0.23217 |  |  |  |  |  |
| 12 | 76->84 | 0.22511 | 7.3371 | 168.98 | 0.0282 | 0.002 | 2.0597 |
|  | 78->85 | 0.53883 |  |  |  |  |  |
| 13 | 79->84 | 0.64785 | 7.4996 | 165.32 | 0.0009 | 1.5849 | 1.6945 |
| 14 | 77->84 | -0.33308 | 7.6652 | 161.75 | 0.0032 | -13.1766 | -11.786 |
|  | 77->85 | 0.43623 |  |  |  |  |  |
|  | 78->85 | -0.26179 |  |  |  |  |  |
| 15 | 82->86 | -0.43383 | 7.7524 | 159.93 | 0.0042 | -4.6263 | -5.1012 |
|  | 83->86 | 0.52233 |  |  |  |  |  |
| 16 | 76->84 | 0.39376 | 7.8012 | 158.93 | 0.0078 | -0.1807 | -0.691 |
|  | 77->85 | -0.2445 |  |  |  |  |  |
|  | 78->84 | -0.26118 |  |  |  |  |  |
| 17 | 81->86 | 0.59129 | 7.9324 | 156.30 | 0.0032 | 8.1302 | 8.3845 |
| 18 | 79->85 | 0.59026 | 7.9610 | 155.74 | 0.0059 | 4.4363 | 4.2597 |
| 19 | 81->90 | 0.28597 | 8.0406 | 154.20 | 0.0107 | -31.4727 | -33.9247 |
| 20 | 81->86 | 0.29452 | 8.0528 | 153.96 | 0.0749 | 56.5542 | 58.4154 |
|  | 83->87 | 0.22768 |  |  |  |  |  |
|  | 83->88 | 0.27361 |  |  |  |  |  |
| 21 | 77->84 | 0.29595 | 8.1052 | 152.97 | 0.0276 | -0.2375 | 0.425 |
|  | 82->86 | -0.22664 |  |  |  |  |  |
|  | 83->86 | -0.27226 |  |  |  |  |  |
| 22 | 77->84 | 0.28927 | 8.1328 | 152.45 | 0.0240 | 22.0881 | 21.6383 |
|  | 82->86 | 0.31402 |  |  |  |  |  |
|  | 83->86 | 0.23856 |  |  |  |  |  |
| 23 | 73->84 | -0.22461 | 8.1833 | 151.51 | 0.0074 | 1.5237 | 2.2303 |
|  | 82->87 | 0.38529 |  |  |  |  |  |
|  | 83->87 | -0.34396 |  |  |  |  |  |
| 24 | 82->87 | 0.35605 | 8.2014 | 151.18 | 0.0165 | 5.7862 | 3.6453 |
|  | 83->88 | 0.23412 |  |  |  |  |  |
| 25 | 77->84 | -0.22728 | 8.2645 | 150.02 | 0.0811 | -72.6762 | -88.8085 |
|  | 77->86 | 0.4618 |  |  |  |  |  |
|  | 78->86 | -0.23602 |  |  |  |  |  |
| 26 | 76->85 | 0.35055 | 8.3192 | 149.03 | 0.0143 | 9.326 | 8.0499 |
|  | 83->89 | 0.3751 |  |  |  |  |  |
| 27 | 76->85 | 0.38536 | 8.3589 | 148.33 | 0.0721 | 58.0405 | 62.6399 |
|  | 83->89 | -0.36362 |  |  |  |  |  |
| 28 | 68->84 | 0.43899 | 8.4224 | 147.21 | 0.0340 | -21.7264 | -22.7803 |
| 29 | 73->85 | 0.27746 | 8.4293 | 147.09 | 0.0147 | -16.1338 | -19.0944 |
|  | 74->85 | 0.4075 |  |  |  |  |  |
| 30 | 80->87 | 0.23511 | 8.5474 | 145.06 | 0.0022 | -4.1707 | -3.8048 |
|  | 82->88 | 0.34259 |  |  |  |  |  |
|  | 83->88 | -0.22474 |  |  |  |  |  |

*^a^*Number of the excited states; *^b^*Only transitions with contribution over 10.0% were listed; *^c^*Configuration-interaction coefficient; *^d^*Excitation energy; *^e^*Wavelength; *^f^*Oscillator strength; *^g^*Rotatory strength in velocity form (10^-40^ cgs); *^h^*Rotatory strength in length form (10^-40^ cgs).

**Table S20.** Key transitions, oscillator strengths, and rotatory strengths in the ECD spectrum of conformer **2-4** at the Cam-B3LYP/Def2SVP level of theory in MeOH with IEFPCM solvent model.

| ***Num^a^*** | ***Transition^b^*** | ***CI-coeff^b^*** | ***ΔE (eV)^d^*** | ***λ (nm)^e^*** | ***f^f^*** | ***R_vel_^g^*** | ***R_len_^h^*** |
| --- | --- | --- | --- | --- | --- | --- | --- |
| 1 | 81->84 | 0.63154 | 3.8060 | 325.76 | 0.0003 | -3.2825 | -2.6395 |
|  | 82->84 | -0.23091 |  |  |  |  |  |
| 2 | 81->85 | 0.23983 | 4.1344 | 299.88 | 0.0006 | 0.2072 | -2.8519 |
|  | 82->85 | 0.59166 |  |  |  |  |  |
| 3 | 83->84 | 0.68206 | 5.1985 | 238.50 | 0.2762 | -10.1684 | -11.9903 |
| 4 | 79->86 | 0.67737 | 5.9593 | 208.05 | 0.0015 | 1.8633 | 2.2975 |
| 5 | 80->84 | 0.31246 | 6.0852 | 203.75 | 0.0060 | 9.7347 | 11.9859 |
|  | 82->84 | 0.56226 |  |  |  |  |  |
| 6 | 80->85 | 0.59124 | 6.2798 | 197.43 | 0.0044 | 4.8718 | 4.3432 |
|  | 82->85 | 0.22531 |  |  |  |  |  |
| 7 | 76->84 | -0.32258 | 6.7836 | 182.77 | 0.0008 | -5.3286 | -5.5279 |
|  | 77->84 | 0.44253 |  |  |  |  |  |
|  | 80->84 | -0.25704 |  |  |  |  |  |
| 8 | 83->85 | 0.6257 | 6.9470 | 178.47 | 0.0068 | 1.2009 | 1.2661 |
| 9 | 74->84 | -0.30456 | 7.0072 | 176.94 | 0.0003 | 4.0709 | 4.3018 |
|  | 80->84 | 0.43275 |  |  |  |  |  |
|  | 82->84 | -0.25244 |  |  |  |  |  |
| 10 | 76->85 | -0.26009 | 7.2124 | 171.90 | 0.0235 | -4.4026 | -4.0666 |
|  | 78->85 | 0.52277 |  |  |  |  |  |
| 11 | 77->85 | -0.23518 | 7.4724 | 165.92 | 0.0019 | -11.5828 | -11.6601 |
|  | 79->85 | -0.27507 |  |  |  |  |  |
|  | 81->85 | 0.42809 |  |  |  |  |  |
| 12 | 69->84 | -0.22705 | 7.5034 | 165.24 | 0.0037 | -8.7487 | -11.3332 |
|  | 74->84 | 0.28734 |  |  |  |  |  |
|  | 80->84 | 0.30126 |  |  |  |  |  |
| 13 | 79->85 | 0.43723 | 7.6375 | 162.34 | 0.0013 | 1.2911 | 1.3927 |
|  | 81->85 | 0.40704 |  |  |  |  |  |
| 14 | 74->84 | 0.28539 | 7.8820 | 157.30 | 0.0236 | 4.3098 | 8.7135 |
|  | 81->88 | 0.30921 |  |  |  |  |  |
|  | 83->88 | -0.22758 |  |  |  |  |  |
| 15 | 75->85 | 0.38355 | 7.9255 | 156.44 | 0.0232 | 10.7347 | 9.7316 |
|  | 77->85 | -0.26171 |  |  |  |  |  |
|  | 78->85 | -0.2498 |  |  |  |  |  |
| 16 | 82->86 | 0.51428 | 7.9365 | 156.22 | 0.0363 | -11.0752 | -15.2177 |
| 17 | 76->84 | 0.29984 | 7.9572 | 155.81 | 0.0657 | -58.7913 | -49.3441 |
|  | 83->87 | 0.22833 |  |  |  |  |  |
| 18 | 75->85 | 0.4366 | 8.0111 | 154.77 | 0.0064 | -3.8799 | -3.9872 |
|  | 77->85 | 0.26916 |  |  |  |  |  |
|  | 79->85 | -0.33579 |  |  |  |  |  |
| 19 | 76->84 | -0.29564 | 8.0472 | 154.07 | 0.0727 | -14.6024 | -7.8285 |
|  | 83->87 | 0.36616 |  |  |  |  |  |
| 20 | 69->84 | 0.45404 | 8.1255 | 152.59 | 0.0108 | -26.7656 | -24.1327 |
|  | 70->84 | 0.22676 |  |  |  |  |  |
| 21 | 78->86 | -0.23295 | 8.1407 | 152.30 | 0.0824 | -40.8354 | -42.7326 |
|  | 82->87 | 0.47846 |  |  |  |  |  |
| 22 | 76->85 | 0.46657 | 8.1836 | 151.50 | 0.0005 | 0.4819 | 2.1004 |
|  | 77->85 | -0.22643 |  |  |  |  |  |
| 23 | 79->84 | 0.63243 | 8.2173 | 150.88 | 0.0088 | -2.1325 | -1.9632 |
| 24 | 83->87 | 0.37489 | 8.2640 | 150.03 | 0.0468 | 54.0801 | 44.4184 |
|  | 83->88 | 0.2776 |  |  |  |  |  |
|  | 83->90 | 0.32363 |  |  |  |  |  |
| 25 | 77->86 | 0.23176 | 8.3235 | 148.96 | 0.0700 | 10.769 | 6.1209 |
|  | 78->86 | 0.43633 |  |  |  |  |  |
|  | 82->86 | -0.25735 |  |  |  |  |  |
|  | 82->87 | 0.23712 |  |  |  |  |  |
| 26 | 71->84 | -0.23965 | 8.4019 | 147.57 | 0.0636 | 16.457 | 16.9264 |
|  | 72->84 | 0.4471 |  |  |  |  |  |
| 27 | 83->88 | -0.30715 | 8.5886 | 144.36 | 0.0047 | 30.9325 | 24.6448 |
|  | 83->89 | 0.43575 |  |  |  |  |  |
|  | 83->90 | 0.24107 |  |  |  |  |  |
| 28 | 71->85 | 0.24064 | 8.6067 | 144.06 | 0.0026 | -0.1365 | -0.0187 |
|  | 73->85 | -0.24502 |  |  |  |  |  |
|  | 74->85 | 0.40094 |  |  |  |  |  |
| 29 | 77->84 | 0.32585 | 8.6310 | 143.65 | 0.0048 | 4.4449 | 4.6712 |
|  | 78->84 | 0.49888 |  |  |  |  |  |
| 30 | 83->86 | 0.64777 | 8.6732 | 142.95 | 0.0016 | -3.8201 | -3.5613 |

*^a^*Number of the excited states; *^b^*Only transitions with contribution over 10.0% were listed; *^c^*Configuration-interaction coefficient; *^d^*Excitation energy; *^e^*Wavelength; *^f^*Oscillator strength; *^g^*Rotatory strength in velocity form (10^-40^ cgs); *^h^*Rotatory strength in length form (10^-40^ cgs).
